# The association between procrastination and negative emotions in healthy individuals: a systematic review and meta-analysis

**DOI:** 10.3389/fpsyt.2025.1624094

**Published:** 2025-10-23

**Authors:** Yuyang Nie, Wenlei Wang, Fangbing Zhou, Tianci Wang, Simin Li, Cong Liu, Jinchao Gao

**Affiliations:** ^1^ College of Physical Education and Sports, Beijing Normal University, Beijing, China; ^2^ College of Education for the Future, Beijing Normal University, Zhuhai, China; ^3^ School of Physical Education, Ningxia University, Yinchuan, China; ^4^ School of Physical Education and Sports Science, Qufu Normal University, Qufu, China

**Keywords:** procrastination, negative emotions, depression, anxiety, stress, systematic review

## Abstract

**Introduction:**

The widespread recognition of the link between procrastination and negative emotions is accompanied by a need for greater clarity regarding the underlying mechanisms of this connection. This study aims to systematically review and meta-analyze the association between procrastination and negative emotions, specifically focusing on depression, anxiety, and stress.

**Methods:**

Through comprehensive searches across five databases, we have included a total of 88 studies, encompassing 63,323 participants across 17 countries. Utilizing Stata 18.0, we conducted separate meta-analyses for each of the three negative emotions.

**Results:**

The results indicate a moderate positive correlation between procrastination and negative emotions, with a combined effect size of r=0.342. Subgroup analyses reveal variations in the strength of this association across different types of procrastination. Furthermore, the results of the publication bias test indicate no significant bias.

**Discussion:**

By unveiling the close connection between procrastination and negative emotions, and preliminarily exploring the bidirectional relationship between procrastination and negative emotions based on the included longitudinal studies, this study has reinforced the theoretical foundation of this field. Policymakers should consider the association with procrastination behaviors when aiming to improve people’s mental health and well-being.

**Systematic Review Registration:** PROSPERO (CRD420251041427).

## 1 Introduction

### 1.1 Prevalence of procrastination

Procrastination is a self-regulatory failure in which an individual voluntarily postpones or procrastinates when faced with a task or responsibility despite anticipating the negative consequences of procrastination (1). It is a prevalent behavior, with research findings suggesting that procrastination exists in approximately 20%-25% of the general population (2). Among young people, procrastination is even more prevalent (3, 4). As represented by students, academic procrastination has also received much attention and has been thoroughly studied. The most common types of academic procrastination include postponement of term paper writing, exam preparation, and usual assignments. Statistics show that up to 70% of college students self-identify as procrastinators (5). A meta-analysis showed that procrastination was significantly associated with decreased academic performance (6). An extensive web-based study by Gröpel and Steel (7) found that procrastination was negatively correlated with age and female gender, i.e., procrastination is likely to decrease with age. However, procrastination still affects academic performance and life satisfaction among contemporary young people (8) and leads to many adverse outcomes (9).

Procrastination is widespread in a number of domains, such as decision-making, exercise, and academic procrastination, of which academic procrastination has been a focus of research. In recent years, the research field of procrastination has expanded from traditional academic behaviors to health behaviors, and bedtime procrastination (10) is one example. As a subtype of procrastination, sleep procrastination has similar predictors to general procrastination. Notably, similar to general procrastination, sleep procrastination has been found to be significantly associated with cell phone addiction (11, 12). This is an expansion of a new predictor variable on procrastination following the meta-analysis of (1). In addition, sleep procrastination is strongly associated with lower self-control, a late bedtime routine, increased use of electronic media, higher state and trait anxiety, and depressive symptoms and tends to lead to decreased sleep quality (13), which in turn negatively impacts mental health.

### 1.2 The association between procrastination and negative emotions

Given the prevalence of procrastination in contemporary society, in addition to the ongoing exploration of predictors of procrastination, there has been a growing body of research on how procrastination affects health behaviors and mental health. An example is the procrastination-health model (14, 15). In addition, previous research has strongly linked procrastination to the Big Five personality traits (16, 17). Neuroticism is also the strongest predictor of procrastination in the Big Five (18). Neuroticism as a personality trait is centrally characterized by susceptibility to and intensity of response to negative emotions (19). Highly neurotic individuals are more likely to experience negative emotions such as anxiety, depression, and stress and have difficulty regulating them effectively (20). Based on this, we hypothesize that negative emotions are important in procrastination behavior. Neuroanatomically dissected, chronic stress and depression lead to hippocampal reduction (21), and the hippocampus plays a key role in individual self-regulation. Furthermore, specific structures in the right hippocampus may form the neural basis of the association between trait anxiety and procrastination (22), which provides a neuroanatomical level of explanation for understanding the material link between procrastination and depression, anxiety, and stress.

In addition, many cross-sectional studies have confirmed the strong association between procrastination and adverse effects (23, 24), further reinforcing the link between procrastination and mental health. Longitudinal studies have further revealed a possible causal relationship between the two. For example, depression, anxiety, and stress are effective predictors of procrastination (25, 26). Similarly, procrastination was also effective in predicting levels of depression, anxiety, and stress at future time points (27, 28). However, some studies have measured the correlation between the two differently (29, 30), which may be due to differences in sample idiosyncrasies and the types of variables involved.

Taking into account the existing literature, there may be a vicious circle between negative emotions and procrastination: On the one hand, negative emotions, such as anxiety, depression, and stress, weaken an individual’s sense of self-efficacy, motivation, and executive functioning, which can lead to the development and maintenance of procrastination behaviors (31). For example, anxiety may lead individuals to avoid tasks, depression may lead to a lack of motivation, and stress may lead to difficulty concentrating. On the other hand, procrastination itself can exacerbate negative emotions, creating a vicious cycle of “procrastination-negative emotions”. The stress, guilt, and self-depreciation associated with not completing a task can further exacerbate negative emotions such as anxiety and depression, creating a self-reinforcing cycle.

In summary, most of existing research supports a positive association between procrastination and negative emotions (depression, anxiety, and stress), and a large body of research tends to support a positive correlation between the two. However, there is a lack of systematic sorting and quantitative synthesis of this relationship. Given this, the present study intends to comprehensively integrate the empirical data on the relationship between procrastination and depression, anxiety, and stress in the existing literature by using systematic review and meta-analysis, aiming to reveal the overall effect size of procrastination behaviors on these three negative emotions and their potential moderating factors and to explore in depth their intrinsic mechanisms of action, to provide evidence-based basis for the development of targeted interventions.

### 1.3 Overview of the main elements of this study

This study explored the association between procrastination and negative emotions through systematic evaluation and meta-analysis. Among other things, negative emotions were mainly measured using depression, anxiety, and stress. These three emotional states are often used to assess psychological distress or negative emotions ( (32–34). First, we counted the extent to which depression, anxiety, and stress were associated with procrastination. We plotted the corresponding forest and funnel plots to visualize the results of the analyses for each dimension. Subsequently, we combined the effect values of the three to assess the overall association between procrastination and negative emotions comprehensively. Next, subgroup analyses were conducted based on different grouping criteria to explore the variability in the strength of the association under different categories. Finally, based on the included longitudinal studies, we preliminarily explored the directional association between procrastination and negative emotions.

## 2 Method

The study utilized a combination of qualitative and quantitative methodologies, including systematic review and meta-analysis, while strictly adhering to the PRISMA guidelines (Preferred Reporting Items for Systematic Reviews and Meta-Analyses). It is registered with PROSPERO under the registration number PROSPERO 2025 CRD420251041427.

### 2.1 Search strategy

Based on the PRISMA statement guidelines, five databases, PubMed, Web of Science, Scopus, ProQuest, and EBSCO, were systematically searched in this study. The search formula used the following keyword combinations: (“procrastination” OR “procrastination behavior” OR “ procrastination tendency” OR “delaying behavior”) AND (“anxiety” OR “anxiety disorder” OR “generalized anxiety disorder” OR “social anxiety disorder” OR “panic disorder” OR “depression” OR “major depressive disorder” OR “clinical depression” OR “dysthymia” OR “stress” OR “stressor” OR “stress response” OR “occupational stress” OR “chronic stress”). The search terms were concatenated using the appropriate operators according to the syntax rules of each database. The search timeframe was limited to March 5, 2025, when each database was constructed. A manual search of Google Scholar was conducted to complement the electronic database searches. All retrieved documents were imported into Zotero software for de-duplication in preparation for further screening.

### 2.2 Inclusion and exclusion criteria

Inclusion criteria for this meta-analysis were as follows: ① cross-sectional, longitudinal, or cohort study design; ② the study had to be a peer-reviewed empirical study; ③ the literature had to report data on the association between delay and depression, anxiety, or stress; such as correlation coefficient r or regression coefficient (β); ④ the study had to be conducted on a healthy population. ⑤ The literature can be published in any country, but must be a journal article published in English.

Exclusion criteria for this meta-analysis were as follows: literature in a language other than English; non-empirical studies, such as reviews, theoretical articles, case studies, etc.; literature that did not report data on the association between delay and depression, anxiety, or stress, e.g., statistics such as correlation coefficients, r, regression coefficients, etc., were not provided; studies of clinically diagnosed patients or disease-specific populations; literature that had duplicate data for publication; abstracts of conferences, dissertations, and non- formally published literature; literature with low quality studies and serious methodological flaws that may lead to unreliable results.

### 2.3 Screen studies and data extraction

The literature retrieved from the database was de-weighted and then entered into a systematic screening process. The process began with an initial skimming of titles to weed out irrelevant literature, followed by reading the abstracts of the remaining literature to further screen for literature that fit the study topic, and finally, the literature screened through the abstracts was reviewed and assessed in full text to finalize the literature to be included in the study. Data extraction was done independently by two authors, and the extracted information included authors, year of publication, country, participant characteristics, procrastination measurement tool, negative mood measurement tool (mainly extracting data related to depression, anxiety, and stress), correlation coefficients, and study conclusions. Among them, the country information was based on the ISO 3166–1 standard issued by the International Organization for Standardization (ISO), which uses country codes for uniform presentation. After the extraction was completed, two authors cross-checked. In case of disagreement, a third researcher made an independent assessment and adjudicated the final results.

### 2.4 Quality assessment

This study used the Mixed Methods Appraisal Tool (MMAT 2018 version) to evaluate the methodological quality of the included quantitative observational studies (35). The MMAT is suitable for quality assessment of a wide range of study designs in systematic evaluations, and its assessment entries for quantitative, non-randomized studies have been rigorously validated to effectively identify potential bias in study design, implementation, and reporting. Two investigators independently assessed five core criteria for each study: sample representativeness, measurement validity, control of confounding, completeness of results, and presence of exposure. Each criterion was scored as 1 point for compliance, with a total score of 0-5. Disagreements were resolved through discussion or third-party arbitration. Literature was ultimately categorized as high (5), fair (4), moderate (3), or low (2 ≥) quality based on scores, and low-quality literature was excluded from sensitivity analyses to validate the stability of results.

### 2.5 Data analysis

In order to ensure the consistency and comparability of the study, in this study, we uniformly used the Pearson correlation coefficient r as the effect size indicator. For the regression coefficient β, we used the conversion formula proposed by Peterson and Brown (36) to convert it to the value of r: r = β × 0.98 + 0.05λ, where λ = −1 when −0.5 < β < 0, and λ = 1 when 0 < β < 0.5. Subsequently, for the meta-analysis, we performed a Fisher’s Z transformation of the correlation coefficient r as follows Fisher’s Z transformation with the following formula: Fisher’s Z = 0.5 * ln [(1 + r)/(1 −r)] with variance Vz = 1/(n−3) and standard error SEz = sqrt [1/(n −3)]. A meta-analysis was then performed using Stata 18.0, with effect sizes between 0.10 and 0.29 considered small, 0.30 and 0.49 considered moderate, and effects above 0.50 considered high (37).

Next, I² was used to determine heterogeneity between studies; if I2 < 50%, heterogeneity between studies was considered acceptable, and a fixed-effects model was chosen for the meta-analysis. If P < 0.1 and I2 > 50%, heterogeneity between studies was considered to exist, and a random effects model was chosen (38). Additionally, if significant heterogeneity was present, subgroup analysis and meta-regression were employed to explore the sources of heterogeneity. Finally, funnel plots and Egger’s test were used to assess publication bias. Publication bias was considered to exist if the p-value of Egger’s test was <0.05 (39).

## 3 Results

### 3.1 Search results

**Figure 1:**
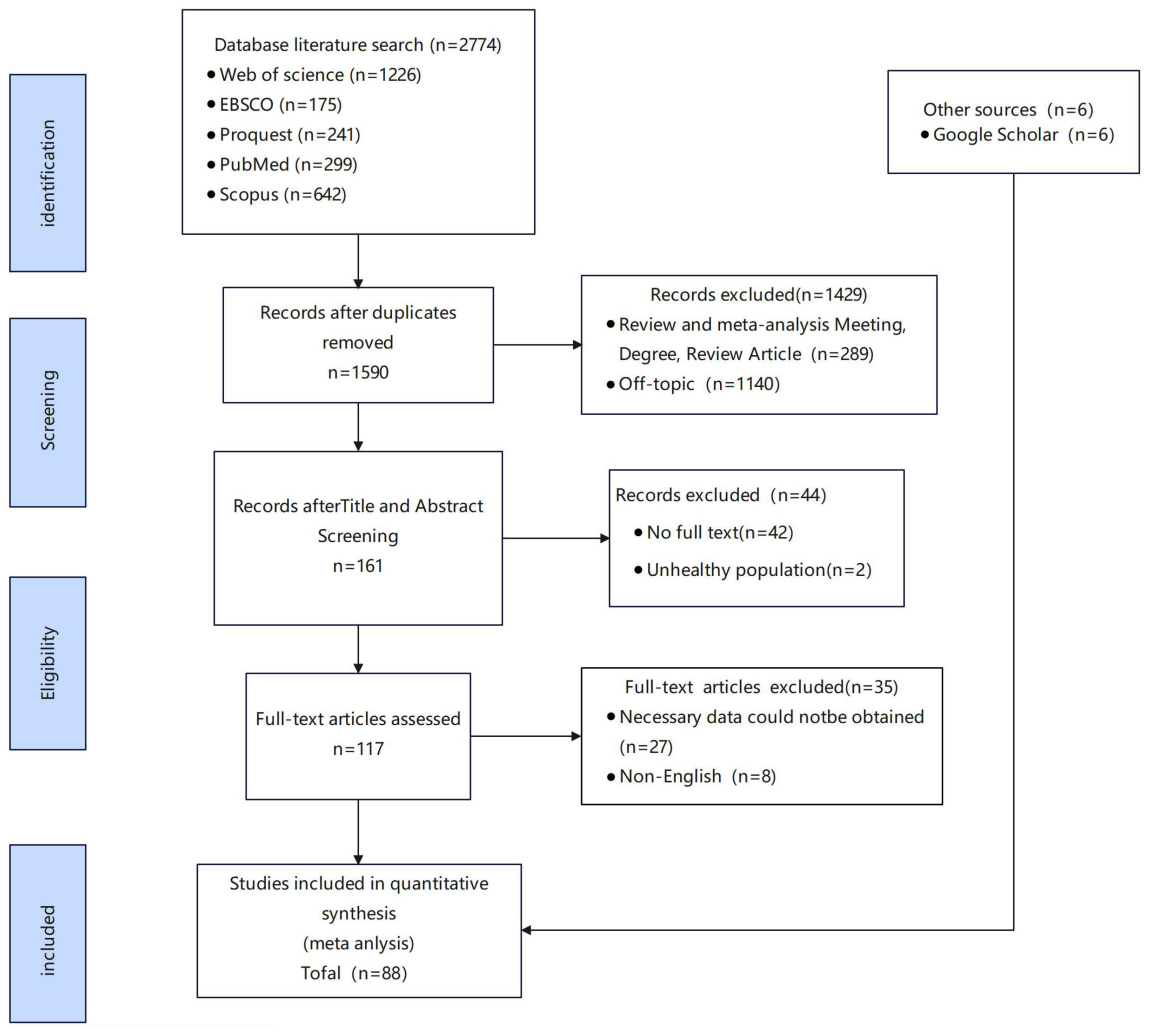
Flow diagram for included studies.

We retrieved a total of 2,744 documents from five databases. After the de-duplication process using Zotero, the number of literature was reduced to 1,590 articles. Further screening revealed that 289 of them were non-research articles. Subsequently, we screened the remaining 1,301 documents for titles and excluded 1,140, leaving 161 for full-text search. During the full-text search, we excluded 42 articles for which full text was unavailable and 2 studies of non-healthy populations, resulting in 117 documents. During the full-text screening stage, we excluded 8 non-English literature and 27 literature that did not report relevant data. In addition, by manually searching for similar keywords on Google Scholar, we added six additional literatures. Ultimately, the meta-analysis included 88 studies that explored the association between procrastination and depression, stress, and anxiety (**Figure 1**).

### 3.2 Description of studies

Supplementary I contains basic information on 88 documents that cover the geographical distribution of 17 countries. All age groups were covered, with college students being the most prevalent at 77.27%. The sample size was 49-4,196, totaling 63,323 individuals. Measurement methods for procrastination and negative emotions relied heavily on self-reports. Procrastination was measured by a wide variety of instruments, including the General Procrastination Scale (GPS), Procrastination Scale for Students (PASS), Academic Procrastination Scale (APS), Adult Procrastination Inventory (AIP), Sleep Procrastination Scale (BPS), and Decision Procrastination Scale (DPS), among others. Measures of negative affect were similarly characterized by diversity, with the Anxiety Depression Stress Scale (DASS-21) being the most widely used. For anxiety assessment, a variety of types were covered, including state anxiety, test anxiety, social anxiety, academic anxiety, generalized anxiety, and cognitive anxiety; for depression measurement, a variety of scales were used, including the CES-D, PHQ-9, BDI-SF, and SRQ-20; and for stress, the measure relied heavily on the Perceived Stress Scale (PSS), with types including academic stress and work stress. All of the literature reported data on the association of procrastination with depression, anxiety, or stress.

### 3.3 Quality assessment results

The 88 quantitative non-randomized studies included in this study were critically assessed for quality. All the literature met the first two basic criteria of MMAT (2018 version) and were recognized as qualified studies. The results of the quality assessment showed that the overall quality of the literature was high, with 32 (36.36%) high-quality, 34 (38.64%) good-quality, 20 (22.73%) moderate-quality, and 2 (2.27%) low-quality literature. Notably, these two low-quality studies were both published before 2000; their common limitations included inadequate sample representativeness and insufficient control of confounding variables, primarily constrained by the underdeveloped reporting standards at that time. Furthermore, sensitivity analysis indicated that after excluding these studies, there were no significant changes in the effect sizes and heterogeneity of the research findings (effect size change < 5%, I² change < 3%). Considering the minimal impact of these two studies on the statistical results of the pooled data and to avoid “time truncation bias,” the research team decided to retain them. Overall, the quality distribution of the included literature was well-balanced, providing a reliable methodological foundation for the findings of this studyD:\Sue\COPY EDIT\FILES\2025\Sep 16 to October 15\10-11\10–11 SUSAN\L2\fpsyt.2025.1624094\Basic Information and Quality Assessment of the Literature.docx.

### 3.4 Meta- analysis results

**Figure 2:**
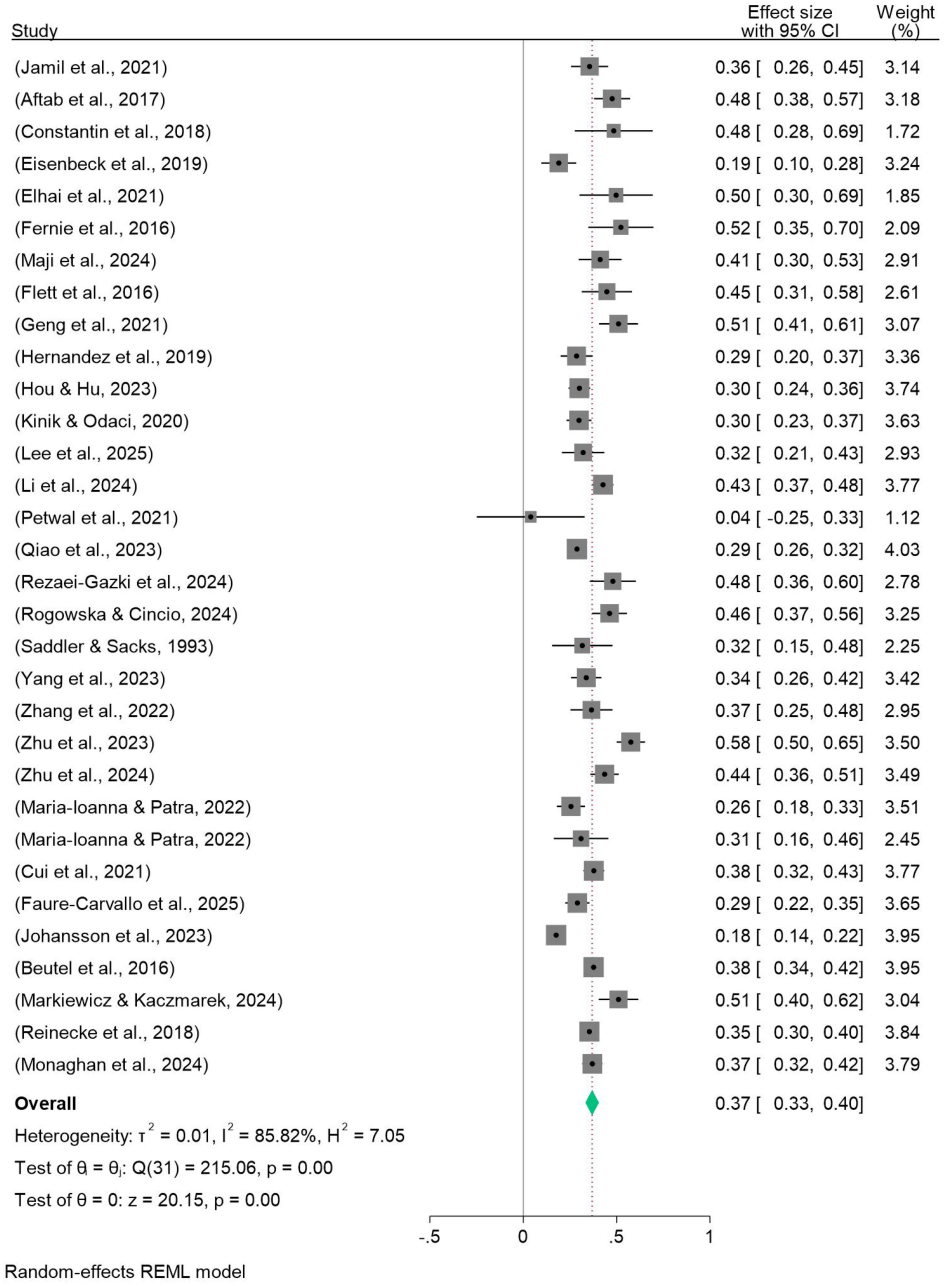
Forest plot of meta-analysis on the relationship between procrastination and depression.

**Figure 3:**
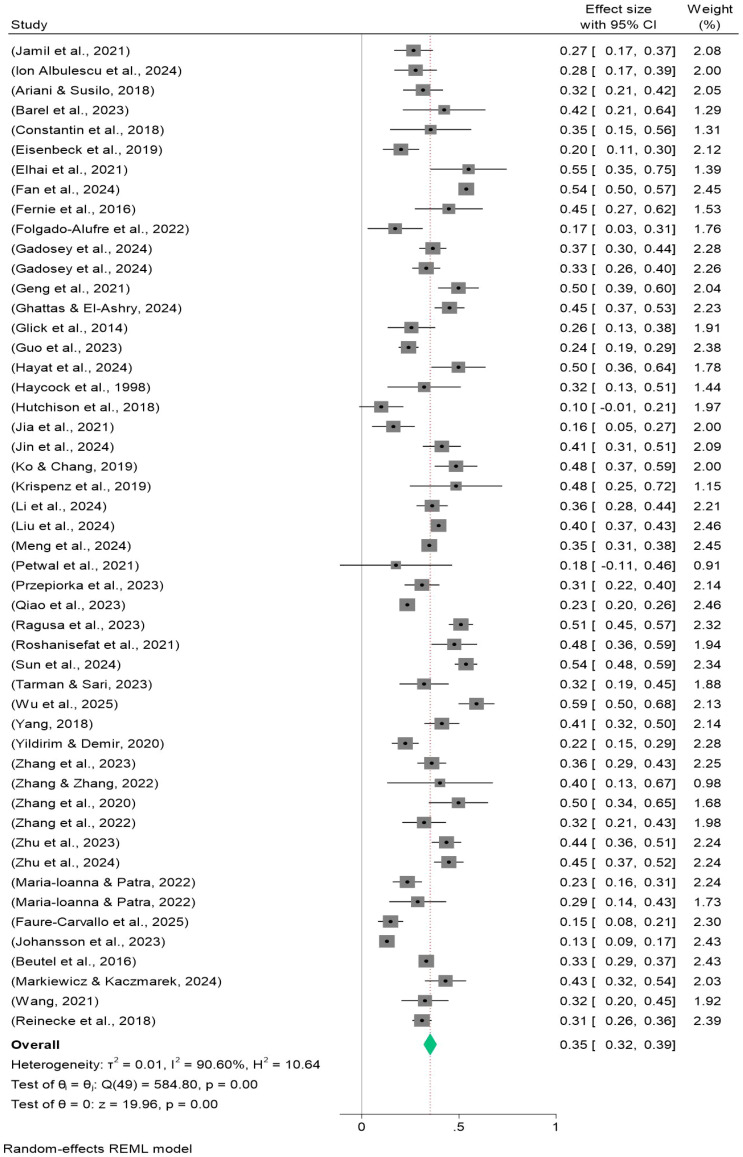
Forest plot of meta-analysis on the relationship between procrastination and anxiety.

**Figure 4:**
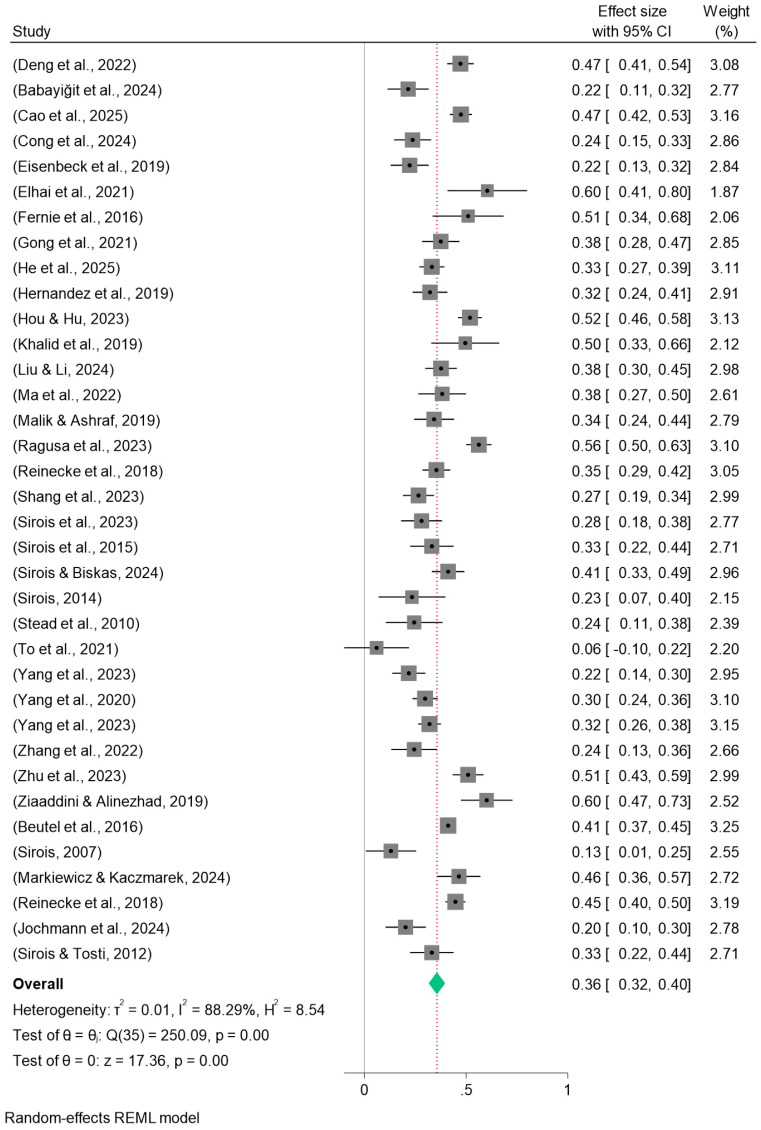
Forest plot of meta-analysis on the relationship between procrastination and stress.

In this study, maximum likelihood estimation (MLE) model and random effects model were used to synthesize and analyze the association between procrastination and depression, anxiety, and stress, and the results are shown in **Table 1**:

- Procrastination was moderately strongly positively correlated with depression (k = 32, Fisher’s Z = 0.369, 95% CI: 0.333-0.405, Q(df) = 215.06(31)), and forest plot results are shown at **Figure 2**. The combined Fisher’s Z was converted to the correlation coefficient, yielding r = 0.353.
- Procrastination was also positively correlated with anxiety at moderate strength (k = 50, effect = 0.352, 95% CI: 0.317-0.386, Q(df) = 584.80(49)), with forest plot results as shown in **Figure 3**. The combined Fisher’s Z was converted to the correlation coefficient, yielding r = 0.338.
- Procrastination was similarly positively correlated with stress at moderate strength (k = 36, Fisher’s Z = 0.357, 95% CI: 0.316-0.397, Q(df) = 256.09(35)), as shown in the forest plot results **Figure 4**. All associations were statistically significant (p < 0.001). The combined Fisher’s Z was converted to the correlation coefficient, yielding r = 0.343.

**Table 1:** Meta-analysis results summary.

Maximum likelihood estimation, MLE				
Model parameter	Depression	Anxiety	Stress	Overall
k	32	50	36	88
Effect (95% CI)	0.369 (0.333-0.405)	0.352 (0.317-0.386)	0.357 (0.316-0.397)	0.356 (0.332-0.379)
Q (df)	215.06 (31)	584.80 (49)	250.09 (35)	688.86 (87)
I²	85.82%	90.60%	88.29%	87.20
τ²	0.0079 (0.0024, 0.0135)	0.014 (0.0065, 0.0216)	0.0103 (0.0058, 0.0147)	0.01 (0.0058, 0.0142)
Z	20.15	19.96	17.36	29.69
n	24755	41516	22037	63,323

After internally combining each study to ensure that only one effect size was included per study, the meta-analysis results showed that the overall effect size was Fisher’s Z = 0.356 (95% CI: 0.332-0.379), indicating a moderate positive correlation between procrastination and negative mood. Heterogeneity tests revealed significant between-study differences, with 85.82% of the variance in effect sizes for procrastination and depression stemming from true differences in effects across studies, and those proportions are 90.6% and 88.29% for anxiety and stress, respectively. In addition, all effect sizes passed the robustness test (Z > 17.36) with p < 0.01, further confirming the robustness of the findings. The combined Fisher’s Z was converted to the correlation coefficient, yielding r = 0.342.

### 3.5 Results of subgroup analysis

To examine the robustness of the main effects, we conducted prespecified subgroup analyses along three dimensions. First, procrastination was classified—based on the instruments employed—into sleep procrastination, general procrastination, academic procrastination, and pure procrastination. Second, studies were dichotomized at 2021, yielding pre-2021 and 2021-and-after cohorts. Third, samples were categorized as non-student adults, secondary-school students, or university students. All comparisons were performed with random-effects models and Q-between tests (P = 0.05). As shown in **Table 2**, the correlation coefficient between procrastination types was 0.332, with a heterogeneity I² of 77.10% for APS and depression, 0.328, with an I² of 91.6% for anxiety, and 0.338, with an I² of 85.40% for stress; the correlation coefficient between GPS and depression was 0.351, with an I² of 69.5%, with an I² of 80% for anxiety, and a stress of 0.297 with an I² of 82.2%; the BPS correlation coefficient with depression was 0.419 with an I² of 87.30%, anxiety was 0.401 with an I² of 66.1%, and stress was 0.4 with an I² of 90.60%; the AIP correlation coefficient with depression was 0.205 with an I² of 62.20%; the PPS correlation coefficient with depression was 0.333 with an I² of 96.1%; and the correlation coefficients of IPS with depression, anxiety, and stress were 0.491 (I² of 0%), 0.506 (I² of 52.20%), and 0.567 (I² of 0%), respectively. Furthermore, the results of between-group differences revealed that subgroups classified by procrastination type exhibited significant differences across all three dimensions: anxiety(P<0.001), depression (P=0.002), and stress (P<0.001).

**Table 2:** Subgroup analysis results.

Group	Depression			Anxiety			Stress		
	r (95% CI)	I²	df	r (95% CI)	I²	df	r (95% CI)	I²	df
Procrastination types grouping									
APS	0.332 (0.273-0.391)	77.10%	8	0.328 (0.261-0.395)	91.6%	18	0.338 (0.254-0.421)	85.40%	7
GPS	0.351 (0.295-0.406)	69.5%	5	0.303 (0.252-0.353)	80%	11	0.297 (0.240-0.355)	82.2%	11
BPS	0.419 (0.333-0.506)	87.30%	5	0.401 (0.362-0.440)	66.1%	6	0.4 (0.312-0.388)	90.60%	5
AIP	0.205 (−0.06-0.47)	62.20%	1						
PPS	0.333 (0.164-0.501)	96.1%	2						
IPS	0.491 (0.348-0.698)	0%	1	0.506 (0.450-0.562)	52.20%	5	0.567 (0.507-0.626)	0%	1
TPS				0.345 (0.098-0.592)	92.40%	1			
Q (P)	26.29 (<0.01)	26.29		151.88 (<0.01)			62.45 (<0.01)		
Year of publication grouping									
2021≤	0.372 (0.326-0.419)	88.4%	21	0.360 (0.315-0.405)	93.3%	31	0.344 (0.288-0.400)	90.%	21
2021>	0.358 (0.306-0.410)	71.4%	9	0.324 (0.272-0.376)	74.5%	14	0.350 (0.300-0.401)	79.6%	14
Q (P)	0.08 (0.778)			0.79 (0.373)			0.06 (0.805)		
Population grouping									
College students	0.369 (0.316-0.422)	87.9%	20	0.352 (0.305-0.399)	91.8%	38	0.332 (0.288-0.376)	84.6%	25
Adult	0.394 (0.281-0.508)	71.9%	3	0.389 (0.314-0.465)	21.9%	2	0.306 (0.084-0.528)	93.2%	4
Middle school students	0.364 (0.279-0.448)	84.7%	4	0.363 (0.257-0.468)	94.3%	5	0.427 (0.307-0.547)	89.4%	3
All age groups	0.369 (0.333-0.405)	0%	1	0.323 (0.293-0.354)	0%	1	0.426 (0.392-0.461)	19.9%	1
Q (P)	0.05 (0.997)			10.93 (0.012)			11.41 (0.01)		

Regarding publication year, the correlation coefficients of procrastination with depression, anxiety, and stress were 0.372 (I² of 88.4%), 0.360 (I² of 93.3%), and 0.344 (I² of 90%), respectively, for studies published in 2021 and before; these correlation coefficients were 0.358 (I² of 71.4%) for studies published after 2021, 0.324 (I² of 74.5%), and 0.350 (I² of 79.6%). The results of the between-group analysis indicated that there were no statistically significant differences in depression (P = 0.778), anxiety (P = 0.373), or stress (P = 0.805) across subgroups stratified by publication year.

In terms of population, the correlation coefficients of procrastination with depression, anxiety, and stress were 0.369 (I² of 87.9%), 0.352 (I² of 91.8%), and 0.332 (I² of 84.6%) for college students, 0.394 (I² of 71.9%), 0.389 (I² of 21.9%), and 0.306 (I² of 93.2%); the correlation coefficients for primary and secondary school students were 0.364 (I² of 84.7%), 0.363 (I² of 94.3%), and 0.427 (I² of 89.4%). The results of the all-age analysis showed that the correlation coefficients of procrastination with depression, anxiety, and stress were 0.369 (I² of 0%), 0.323 (I² of 0%), and 0.426 (I² of 19.9%), respectively. The between-group analysis revealed statistically significant differences in anxiety (P = 0.012) and stress (P = 0.010) among subgroups stratified by age group, whereas no significant difference was observed in depression (P = 0.997). It should be noted that although significant differences exist between subgroups, the high heterogeneity within each subgroup suggests that caution should be exercised when interpreting the results.

### 3.6 Meta-regression analysis

To determine whether the observed between-study heterogeneity could be explained by measurement instrument, sample size, or year of publication, we conducted random-effects meta-regressions with the Knapp–Hartung modification, using the pooled correlation coefficients between procrastination and each negative-emotion domain (depression, anxiety, and stress) as dependent variables. Three covariates were examined: procrastination scale type, sample size, and publication year (**Table 3**).

**Table 3:** Meta-regression results based on procrastination scale, publication year, and sample size.

Moderating Variable	Depression				Anxiety				Stress			
	Coefficient (95% CI)	SE	P	K	Coefficient (95% CI)	SE	P	K	Coefficient (95% CI)	SE	P	K
Procrastination scale	0.020 (−0.015,0.055)	0.017	0.250	32	0.020 (− 0.019,0.059)	0.019	0.311	36	0.010 (−.033,0.054)	0.025	0.636	36
Sample size	−0.297 (−0.542,−0.05)	0.12	0.019	32	−0.110 (− 0.323,0.102)	0.106	0.301	36	0.403 (−0.074,0.880)	0.277	0.095	36
Publication year	0.007 (−0.031,0.045)	0.019	0.712	32	0.039 (−0.013, 0.09)	0.026	0.140	36	0.026 (−0.021,0.072)	0.026	0.274	36
τ² (95% CI)	0.0069 (0.0021, 0.0105)				0.0124 (0.0059, 0.0160)				0.0118 (0.0053, 0.0163)			
Marginal R-squared	0.142				0				0.062			
Total R-squared	0.852				0.906				0.882			
VIF	1.166				1				1.066			

Procrastination scale type did not significantly moderate any of the three outcomes (depression: β= 0.020, SE = 0.017, p = 0.250; anxiety: β= 0.020, SE = 0.019, p = 0.311; stress: β= 0.010, SE = 0.025, p = 0.636). This suggests that differences in effect sizes across instruments (e.g., GPS, APS, BPS) are largely attributable to random variation.

Sample size exerted a significant negative influence only for the depression model (β = −0.297, SE = 0.120, p = 0.019), indicating that larger samples yielded smaller correlations—a pattern consistent with small-study inflation. No significant effects were observed for anxiety or stress (both p > 0.0.05). Sensitivity analysis after excluding studies with extreme sample sizes retained 26 studies (k = 26). The pooled Fisher’s Z was 0.381 (95% CI 0.344–0.417), with τ²= 0.0066 and I² = 79.53%.

Relative to the full sample (Fisher’s Z = 0.369), the point estimate remained virtually unchanged (+3.3%), whereas heterogeneity decreased modestly (τ² declined by 18.5% and I² by ~6 percentage points). These findings indicate that the overall association is robust and is not driven by extreme sample sizes.

Publication year was not associated with effect-size magnitude for any outcome (all p = 0.140–0.712), implying no systematic temporal trend over the past decade. Taken together, the examined covariates account for only a modest proportion of the overall heterogeneity; further research should incorporate additional moderators such as cultural context, age composition, and measurement reliability.

### 3.7 Publication bias

**Figure 5:**
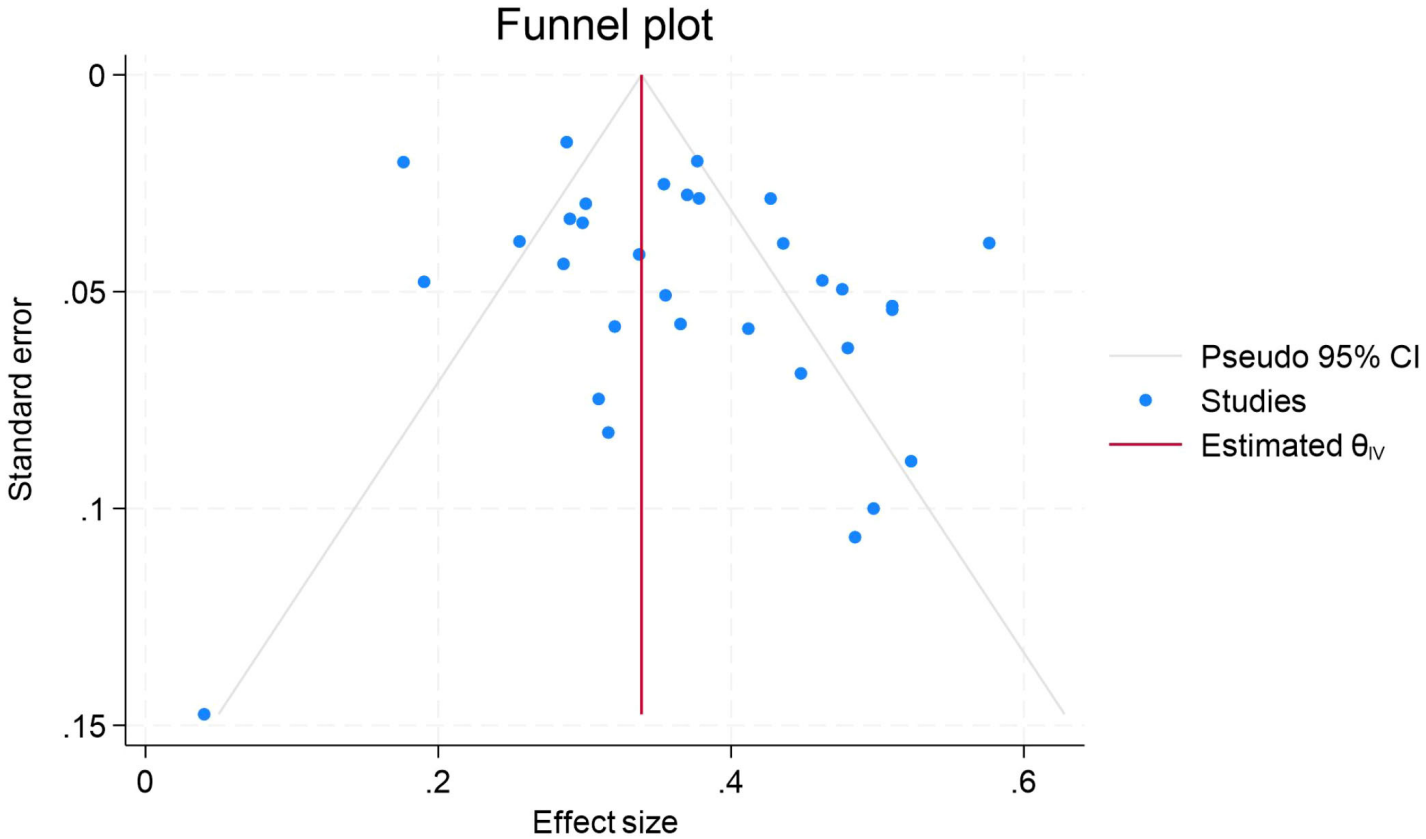
Forest plot of meta-analysis on the relationship between procrastination and depression.

**Figure 6:**
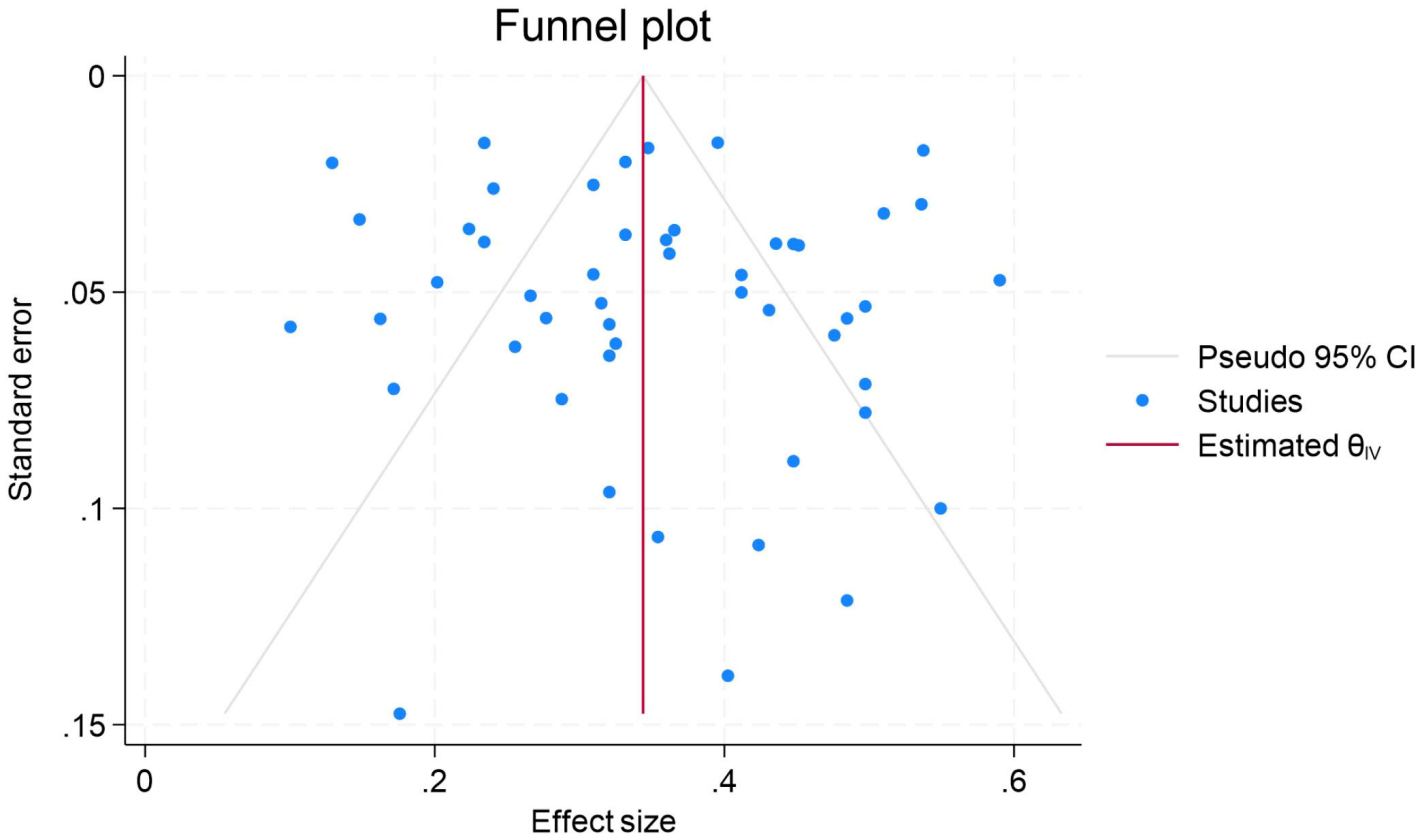
Funnel plot of meta-analysis on the relationship between procrastination and anxiety.

**Figure 7:**
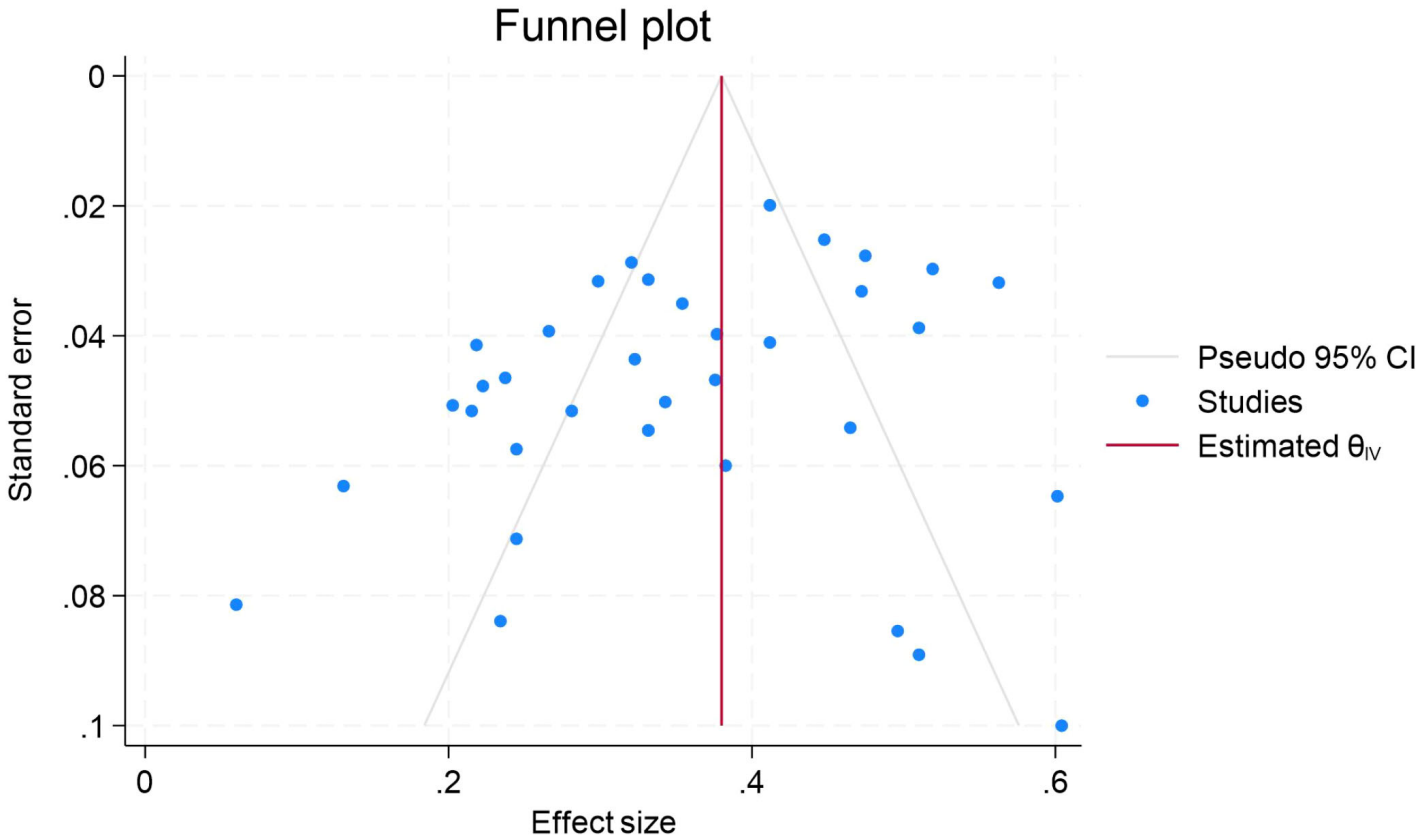
Funnel plot of meta-analysis on the relationship between procrastination and stress.

We conducted publication bias tests on the data of the association of procrastination with depression, anxiety, and stress, respectively. The results of Egger’s test showed that the β-value of procrastination and depression was 0.84, the standard error (SE) was 0.779, and the P-value was 0.2828, and that the β-value of procrastination and anxiety was 0.42, the SE was 0.672, and the P-value was 0.5388. The beta value of procrastination and stress was −0.91, SE was 1.129, and P value was 0.4206. The P values of all three were greater than 0.05, and combined with the symmetry observation of the funnel plots (**Figures 5**-**7**), we can conclude that the results of meta-analyses of procrastination and depression, anxiety, and stress did not find any significant publication bias.

## 4 Discussion

The relationship between procrastination and depression, anxiety, and stress has been a hot topic of research, and the number of related studies has been on the rise in recent years. However, a systematic quantitative summary is lacking. To fill this research gap, the present study used systematic evaluation and meta-analysis to comprehensively analyze the relevant literature. A total of 88 papers containing 118 effect sizes, including 32 for depression, 50 for anxiety, and 36 for stress, were included in this study, involving a total of 63,323 participants and 17 countries. This study provides an in-depth exploration of the relationships between different types of procrastination and depression, anxiety, and stress, while also comparing effect size differences across studies published in different years. Additionally, it examines population differences and potential moderating factors. Meta-analytic findings indicated a significant positive association between procrastination and negative affect (r = 0.342); moreover, the effect size remained stable across publication years, and no evidence of publication bias was detected (Egger’s test, p > 0.05). Nevertheless, this correlation explains only approximately 12% of the variance, leaving nearly 88% unaccounted for. Consequently, the practical magnitude of this “medium” effect may be overestimated in clinical or educational contexts, underscoring the need for future research to systematically examine contextual, personality, and methodological moderators.

### 4.1 Overall association between procrastination and negative emotions

The present study further confirmed the moderate association between procrastination and depression, anxiety, and stress through meta-analysis. This is supported by emotion regulation theory, where procrastination is often accompanied by a failure of emotion regulation, which in turn leads to more negative emotions (40, 41). Notably, this meta-analysis included several longitudinal studies that provide some clues for inferring directionality. For example, some longitudinal studies suggest that procrastination predicts subsequent negative emotions (27, 28, 42, 43). Moreover, other longitudinal studies have shown that negative emotions similarly predict subsequent procrastination (25, 26, 44). However, it is important to emphasize that these longitudinal studies do not completely prove causality because there may be other confounding variables, such as individuals’ personality traits, life circumstances, etc. Therefore, although the results of meta-analyses combined with longitudinal studies enhance our understanding of the relationship between procrastination and negative emotions, caution is still needed in interpreting these results to avoid over-inferring causality. More rigorously designed experimental studies are needed in the future to further explore the causal relationship between procrastination and negative emotions.

Self-Difference Theory (SDT) provides a valuable framework for understanding this relationship. According to the theory, procrastination can be viewed as a manifestation of an individual’s failure to live up to his or her ideal self or should-be self, a disparity that triggers negative emotions and further exacerbates procrastination behavior. A meta-analysis (45) suggests an association between self-discrepancy and psychopathology, which further supports the potential of SDT as a transdiagnostic framework. From an action cybernetics perspective, the experience of positive emotions reduces behavioral inhibition and facilitates the implementation of goal intentions; thus, individuals high in positive affect are less likely to fall into procrastination (44). On the other hand, when individuals are confronted with negative emotions and lack effective emotion regulation strategies, they may choose procrastination as a coping mechanism to temporarily escape these negative emotions (40). The hippocampal-prefrontal circuit plays a pivotal role in both emotional regulation and executive control. Chronic states of depression, anxiety, and stress co-occur with structural and functional alterations in this circuit, and these neural markers are concurrently associated with procrastination tendencies (22). Consequently, high neuroticism, self-discrepancy, and neural circuit characteristics may independently or synergistically compromise an individual’s emotional regulation efficacy, thereby increasing the likelihood of procrastination behaviors. Conversely, the uncompleted tasks and diminished self-evaluation resulting from procrastination may further exacerbate levels of depression, anxiety, and stress, thus establishing a bidirectional or cyclical relationship. It should be emphasized that this framework is intended to illustrate multiple pathways rather than presuppose any specific causal direction.

As highlighted by Pérez-Jorge et al. (9), “tomorrow never comes,” and overcoming procrastination can help improve various unhealthy habits and effectively alleviate mental health issues. Therefore, in addressing student mental health concerns, educational institutions should enhance the prevention and intervention of procrastination behaviors through the implementation of systematic procrastination screening and the establishment of daily plan execution monitoring mechanisms, distinguishing between sleep procrastination and academic procrastination, thereby enabling timely identification and effective management of various factors affecting students’ psychological well-being. Concurrently, healthcare professionals should explore the intrinsic relationship between procrastination and mental health in clinical practice, integrating cognitive behavioral therapy with neuroscientific perspectives to provide more precise and personalized intervention services for diverse populations.

### 4.2 Association of procrastination with specific emotions

In the separate meta-analyses we conducted, no significant difference in the association of procrastination with depression, anxiety, and stress was observed. This may be due to the fact that depression, anxiety, and stress overlap in terms of symptoms. For example, symptoms such as insomnia, poor concentration, and irritability may occur in all three states simultaneously. In addition, anxiety at a higher level may be responsible for a variety of other psychosocial states (46). Stress is a basic short-term problem that can lead to anxiety if left untreated. Anxiety is a chronic problem that may become the cause of major depression. In this vicious circle, depression sometimes leads to anxiety (47).

In addition, separate subgroup analyses for depression, anxiety, and stress revealed variability in the effect sizes by procrastination type. In the anxiety and stress models, subgroup effect sizes were higher for academic and sleep procrastination than general procrastination. However, this difference was not observed in the depression model. The possible reasons for this phenomenon are closely related to the characteristics of the study population. The main participants of this study were the student population, who face heavy academic tasks and high academic pressure, and the probability of anxiety and stress is higher than that of depression (48).

Meanwhile, anxiety related to exams, as well as future planning, further strengthens the link between procrastination behavior and mental health problems. The direct effects of anxiety and stress may interfere with the quality of study and sleep of university students. Therefore, academic and sleep procrastination are more relevant to the student population than procrastination in general. This specificity was similarly observed in a meta-analysis of student procrastination and cell phone addiction (12). These findings suggest that developing customized interventions for specific types of procrastination behaviors (e.g., academic procrastination and sleep procrastination) may be more effective in alleviating mental health problems among students.

### 4.2 Heterogeneity between studies

In the present study, we subgrouped according to procrastination type, publication year, and population to explore heterogeneity. In the procrastination type subgroup, heterogeneity was somewhat explained in the depression, anxiety, and stress models, but the subgroups of publication year and population did not explain any heterogeneity. Notably, the procrastination type subgroup, even if partially explained, remained unexplained for the large remaining heterogeneity. Self-reported procrastination behavior may be influenced by multiple sources of heterogeneity that may lead to inconsistent and difficult-to-interpret findings (49, 50). Similarly, the self-reported validity of different depression and stress measurement instruments and the association of anxiety type with specific types of delay may also have implications for interstudy heterogeneity. Future research needs to consider these potential confounders and use more objective measures, such as behavioral observations or physiological indicators, to more accurately assess procrastination behaviors and their relationship to mental health.

### 4.3 Limitations and future directions

Although the present study revealed a significant overall correlation between procrastination and negative affect and covered a wide range of geographical areas, the available evidence does not clarify the causal relationship between the two. First, the limited number of longitudinal studies makes it difficult to establish the direction of causality, and although the hypothesis that procrastination induces negative affect is somewhat plausible and vice versa, the numerous possible confounders in between have not been adequately controlled for. Second, existing studies have relied mainly on self-report measurement instruments, susceptible to individual answering habits and subjective recall bias. Thirdly, the study sample predominantly comprised university students, with limited representation from other age groups (such as elderly and middle-aged individuals), and the exclusion of non-healthy populations restricts the generalizability of the findings. Finally, the omission of unpublished literature and dissertations may introduce publication bias, potentially compromising the comprehensiveness and reliability of the research conclusions.

In the future, more high-quality longitudinal studies can be conducted, combining statistical methods such as cross-lagged modeling or structural equation modeling and controlling for more potential confounding variables, in order to clarify the causal relationship between procrastination and negative emotions and its direction more clearly. Second is to adopt objective measurement methods such as accelerometers and ecological momentary assessment, reducing the reliance on self-reporting while integrating with longitudinal research to improve the objectivity of the study. Third is to expand the diversity and representativeness of samples, extending research to different populations and cultural backgrounds to enhance the generalizability and applicability of the findings. Finally, based on the in-depth understanding of the mechanisms, is to develop and evaluate effective intervention strategies targeting procrastination and negative emotions and to explore the application of new technologies and methods in the study.

## 5 Conclusion

In order to more systematically summarize the correlations between procrastination and negative emotions (anxiety, depression, and stress), this study quantitatively combined 118 effect sizes, of which procrastination was meta-analyzed with depression (32), anxiety (50), and stress (36). The results of the meta-analysis showed that procrastination was moderately positively associated with depression, anxiety, and stress overall. This study reveals for the first time a specific association between procrastination and these negative emotions. At the same time, the study emphasized the importance of avoiding negative emotions by preventing procrastination and reducing procrastination behaviors through emotion regulation. However, this study also has some limitations in that data collection relied heavily on self-report, which poses some challenges in accurately reflecting information about the subjects.

## Data Availability

The original contributions presented in the study are included in the article/**Supplementary Material**. Further inquiries can be directed to the corresponding authors.
