# Supplementary material for: The association between procrastination and negative emotions in healthy individuals: a systematic review and meta-analysis
Source: Front Psychiatry. 2025 Oct 23;16:1624094. doi: 10.3389/fpsyt.2025.1624094 (PMC12588926; doi:10.3389/fpsyt.2025.1624094)
Supplement: Supplementary file 1 [file SupplementaryFile1.docx]

Table1 Basic Information of the Literature.

| Author year | Study design | Country | Sample Size | **Population** | Ptool | Ngeative emotion  tool | r OR β | Conclusion |
| --- | --- | --- | --- | --- | --- | --- | --- | --- |
| [(Jamil et al., 2021)](#Jamil2021) | CS | IR | 390 | College Students | PPS | DASS-21 | A:r=0.264  D:r=341 | Perfectionism can influence procrastination through anxiety and depression. |
| [(Deng et al., 2022)](#Deng2022) | CS | CN | 913 | College Students | BPS | DASS-21 | S:r=0.44 | Interventions can reduce anxiety and stress to help college students with COVID-19 stress avoid bedtime procrastination. |
| [(Aftab et al., 2017)](#Aftab2017) | CS | US | 412 | College Students | TPS | CES-D | D:r=0.443 | Procrastination does not directly affect depressive emotions, but rather indirectly triggers depression entirely through the mediating factor of insufficient and defective self-control. |
| [(Ion Albulescu et al., 2024)](#Ion2024) | CS | RO | 322 | College Students | APS | CTAS-SF | A:r=0.27 | Cognitive test anxiety plays a mediating role between academic procrastination, subjective well-being, and academic performance. |
| [(Ariani & Susilo, 2018)](#Ariani) | CS | ID | 365 | College Students | APS | Test Anxiety | A;r=0.305 | Self-efficacy mediates the relationship between test anxiety and procrastination. |
| [(Babayiğit et al., 2024)](#Babayigit) | CS | TR | 379 | College Students | APS | SGSS | S;r = 0.212 | Academic procrastination is significantly positively correlated with self-imposed stress and is also associated with an increased risk of complex and conscious sleep bruxism. |
| [(Barel et al., 2023)](#Barel2023) | CS | IL | 88 | College Students | GPS | STAI-S | A;r = 0.40 | Lower testosterone levels strengthen the link between anxiety and procrastination, while higher levels weaken it. |
| [(Cao et al., 2025)](#Cao2025) | CS | CN | 1308 | College Students | APS | PSS | S：r=0.442 | perceived stress is significantly associated with academic procrastination, with positive and negative emotions serving as partial mediators |
| [(Cong et al., 2024)](#Cong2023) | CS | CN | 466 | College Students | BPS | PSS | S：r=0.233 | Bedtime procrastination mediates the relationship between academic stress and sleep quality. |
| [(Constantin et al., 2018)](#Constant2018) | CS | CA | 91 | College Students | IPS | DASS-21 | A:r=0.34  D:r=0.45 | When students experience anxiety and depression, they may engage in procrastination due to excessive rumination on past negative thoughts。 |
| [(Eisenbeck et al., 2019)](#Eisenbeck2019) | CS | ES | 442 | College Students | PASS | DASS-21 | D:r=0.188  A:r=0.199  S:r=0.219 | Psychological inflexibility mediates the relationship between general psychological distress and procrastination. |
| [(Elhai et al., 2021)](#Elhai2021) | CS | US | 103 | College Students | IPS | DASS-21 | D:r=0.46  A:r=0.5  S:r=0.54 | Higher levels of depression, anxiety, and stress symptoms are associated with increased procrastination and surface-level learning。 |
| [(Fan et al., 2024)](#Fan2024) | LS | CN | 3370 | College Students | AP | STAI | A：r=0.491 | State anxiety directly predicts academic procrastination and has a stronger impact on males. Self-depletion has a more significant effect on academic procrastination in females. |
| [(Fernie et al., 2016)](#Fernie2016) | CS | UK | 129 | Adult | DPS | DASS21 | D:r=0.48  A:r=0.42  S:r=0.47 | Decisional procrastination is significantly correlated with negative impacts, all measured metacognitive factors, and all attention control factors。 |
| [(Maji et al., 2024)](#Maji2024) | CS | US | 295 | College Students | GPS | BDI-SF | D:r=0.39 | **Positive procrastination and traditional procrastination both positively predict depression.** |
| [(Flett et al., 2016)](#Flett2016) | CS | US | 214 | College Students | PASS | DASS21 | D：r=0 42 | Procrastination is a moderate predictor of depression. |
| [(Folgado-Alufre et al., 2022)](#Folgado2022) | CS | ES | 194 | College Students | APS | SAS | A:r=0.17 | Intrinsic motivation does not directly affect SA, as its influence is mediated through the use of self-regulation strategies and procrastination. |
| [(Gadosey et al., 2024)](#Gadosey2024) | LS | DE | 789 | College Students | APS | AEQ | A:r=0.35 | Academic procrastination at Time 1 positively predicted study-related anxiety at Time 2. |
| [(Gadosey et al., 2024)](#Gadosey2024（2）) | CS | DE | 744 | College Students | APS | AEQ | A：r=0.32 | **Females are more likely to be anxiety-dominant. Anxiety-dominant students report higher procrastination scores, while hope-dominant students report lower ones.** |
| [(Geng et al., 2021)](#Geng2021) | CS | CN | 355 | College Students | BPS | DASS-21 | D:r=0.47  Β=0.36  A:r=0.46  Β=0.35 | Bedtime procrastination partially mediates the relationship between smartphone addiction and depression. |
| [(Ghattas & El-Ashry, 2024)](#Ghattas2024) | CS | CN | 654 | College Students | APS | AAS | A:r=0.423 | Reducing anxiety, correcting maladaptive behaviors, and enhancing adaptive cognitive and emotional regulation strategies can effectively decrease academic procrastination. |
| [(Glick et al., 2014)](#Glick2014) | CS | US | 258 | College Students | PASS | STAI | A:r=0.25 | Procrastination is positively correlated with anxiety and negatively correlated with psychological flexibility. |
| [(Gong et al., 2021)](#Gong2021) | CS | CN | 460 | College Students | Single question | PSS | S:r=0.359 | Procrastination plays a mediating role between perceived stress and Internet addiction. |
| [(Guo et al., 2023)](#Guo2023) | CS | CN | 1476 | Middle School Students | APS | AEQ | A:r=0.-0.126 | Authoritarian applicants who are devout students experience more anxiety, which in turn leads to more academic procrastination. |
| [(Hayat et al., 2024)](#Hayat2024) | CS | IR | 200 | College Students | APS | Test Anxiety | A:R=0.46 | Procrastination leads to self-handicapping and test anxiety among students, which in turn promotes further self-handicapping. |
| [(Haycock et al., 1998)](#Haycock1998) | CS | US | 111 | College Students | procrastination inquiry | SA  Test Anxiety | A:r=0.31  r=0.23 | Anxiety is positively correlated with procrastination. |
| [(He et al., 2025)](#He2025) | CS | CN | 1021 | College Students | BP | PSS | S:r=0.32 | Stress is positively correlated with bedtime procrastination, and life history strategy plays a mediating role between them. |
| [(Hernandez et al., 2019)](#Hernandez2019) | CS | CL | 529 | Middle School Students | procrastination inquiry | PSS  BDI | S：r=0.412  D：r=0.278 | - **Procrastination can exacerbate stress, depression, and anxiety, thereby increasing the risk of internet addiction.**。 |
| [(Hou & Hu, 2023)](#Hou2023) | CS | CN | 1136 | College Students | BPS | PSS  BDI-II | S：r=0.477  D：r=0.292 | Depression is positively correlated with sleep procrastination, mediated by rumination and mobile phone use. |
| [(Hutchison et al., 2018)](#Hutchison2018) | CS | CA | 300 | College Students | GPS | GADQ-IV | A：r=0.10 | The findings are inconsistent with previous research on the relationship between procrastination and generalized anxiety disorder (GAD). This may indicate that there is no direct link between procrastination and these anxiety disorders, or that such a link is influenced by other factors. |
| [(Jia et al., 2021)](#Jia2021) | CS | CN | 320 | College Students | GPS | Academic Anxiety | A:r=0.161 | The association between academic anxiety and self-handicapping is partially mediated by the level of procrastination |
| [(Jin et al., 2024)](#Jin2024) | CS | CN | 402 | College Students | PASS | Academic Anxiety | A：r=0.39 | Academic procrastination mediates the relationship between smartphone distraction and academic anxiety. |
| [(Khalid et al., 2019)](#Khaid2019) | LS | CN | 140 | College Students | PASS | PSS | S：r=0.459 | Procrastination can increase stress among young adults. |
| [(Kinik & Odaci, 2020)](#Kinik2020) | CS | CN | 862 | College Students | AP | BDI | D:r=0.29 | Self-esteem partially mediates the relationship between depression and academic procrastination. |
| [(Ko & Chang, 2019)](#Ko2019) | CS | CN | 321 | College Students | GPS | SIAS | A:r=0.45 | Social anxiety partially mediates the relationship between resilience and procrastination. |
| [(Krispenz et al., 2019)](#Krispenz2019) | LS | DE | 71 | College Students | APS | Test Anxiety  (STAI-SKD） | A:=0.45 | Test anxiety is positively correlated with procrastination. |
| [(Kühnel et al., 2023)](#Kühnel2023) | LS | DE | 108 | Adult | TPS | Day-specific time pressure | S：r=-0.15 | In the short term, time pressure can facilitate action initiation and prevent procrastination. However, in the long term, sustained time pressure may lead to negative consequences such as resource depletion and decreased positive emotions. |
| [(Lee et al., 2025)](#Lee2025) | CS | KR | 300 | Middle School Students | BPS | PHQ-9 | D：r=0.31 | Among South Korean high school students, greater bedtime procrastination is significantly associated with higher levels of depression, partially mediated by personality traits. |
| [(Li et al., 2024)](#Li2024) | CS | CN | 1233 | College Students | AP | PHQ-9 | D：β=0.36 | Physical activity alleviates academic procrastination through both the mediating effects of high self-esteem and low depression levels, as well as the sequential mediating effects of high self-esteem followed by low depression levels. |
| [(Li et al., 2024)](#Lix2024) | CS | CN | 595 | College Students | PASS | GAD-7 | A;r=0.347 | Resilience and social support are important factors in reducing the impact of academic procrastination on anxiety symptoms among medical students. |
| [(Liu et al., 2024)](#Liu2024) | CS | CN | 4196 | Adult | BPS | GHQ-20 | A:r=0.376 | Alleviating pre-sleep anxiety can help suppress bedtime procrastination behavior. |
| [(Liu & Li, 2024)](#Liu20242) | LS | CN | 636 | College Students | PASS | Academic stress | S：r=0.36 | Academic stress among college students not only directly predicts academic procrastination but also exacerbates procrastination behavior by increasing problematic TikTok use. |
| [(Ma et al., 2022)](#Ma2022) | CS | CN | 281 | College Students | APS | PSS | S：R=0.365 | Perceived stress is an important positive predictor of academic procrastination, with self-regulated learning efficacy and self-control moderating the relationship between stress perception and academic procrastination. |
| [(Malik & Ashraf, 2019)](#Mailk2019) | CS | PK | 400 | College Students | APS | ASS  Academic Stress Scale | S：r=0.33 | There is a significant positive correlation between academic procrastination and academic stress. |
| [(Meng et al., 2024)](#Meng2024) | CS | CN | 3599 | College Students | BPS | DASS-21 | A：r=0.334 | Physical exercise and anxiety play a chain mediating role between smartphone addiction and bedtime procrastination. |
| [(Petwal et al., 2021)](#Petwal2021) | CS | IR | 49 | Adult | AIP | OASIS  BDI | A:r=0.174  D：r=0.04 | Individuals with anxiety disorders report elevated levels of procrastination and lower self-compassion. Self-compassion and decisive procrastination are significantly negatively correlated. |
| [(Przepiorka et al., 2023)](#Przepiorka2023) | CS | PL | 478 | College Students | GPS  DPS | Future Anxiety | A：  GPS:r=0.15  DPS:R=0.45 | Procrastination leads to anxiety about the future, which in turn further leads to problematic new media use. |
| [(Qiao et al., 2023)](#Qiao2023) | LS | CN | 4156 | Middle School Students | GPS | GAD-7  PHQ-9 | A:r=0.23  D:r=0.28 | The impact of the COVID-19 pandemic can influence procrastination behavior among Chinese adolescents by triggering symptoms of anxiety and depression. |
| [(Ragusa et al., 2023)](#Ragusa2023) | CS | ES | 991 | Middle School Students | IPS | Academic  Stress and Anxiety | A：r=0.47  S:r=0.51 | Procrastination is positively correlated with academic stress and anxiety, meaning that the more severe the procrastination, the higher the levels of academic stress and anxiety. |
| [(Reinecke et al., 2018)](#Peinecke2018) | CS | DE | 818 | Middle School Students | GPS | PSS | S：r=0.34 | Procrastination leads to higher perceived stress and lower sleep quality among adolescents. |
| [(Rezaei-Gazki et al., 2024)](#Rezaei2024) | CS | IR | 255 | College Students | PASS | GHQ-28 | D：β=0.404 | Interventions that reduce anxiety may be beneficial in reducing academic procrastination, thereby improving students' academic performance. |
| [(Rogowska & Cincio, 2024)](#Rogowska2024) | CS | PL | 448 | Adult | PPS | PHQ-9 | D:Β=0.39 | TikTok use mediates the relationship between procrastination and depression, with procrastination leading to problematic TikTok use (PTTU), which in turn increases depressive symptoms. |
| [(Roshanisefat et al., 2021)](#Roshanisefat2021) | CS | IR | 281 | College Students | TPS | STAS | A;r=0.443 | The higher the level of test anxiety in students, the greater the degree of academic procrastination. This suggests that test anxiety may exacerbate students' procrastination behavior. |
| [(Saddler & Sacks, 1993)](#Saddler1993) | CS | US | 150 | College Students | AIP | BDI | D：r=0.306 | The tendency of students to procrastinate on academic tasks is positively correlated with depressive symptoms. |
| [(Shang et al., 2023)](#Shang2023) | CS | CN | 651 | College Students | procrastination inquiry | PSS | S:r=0.26 | Perceived stress leads to ego depletion, which in turn prompts civil servants to engage in active procrastination. |
| [(Sirois et al., 2023)](#Sirois2023) | LS | CA | 379 | College Students | GPS | DHS | S:r=0.274 | Procrastination indirectly affects health problems through stress, but the mediating role of health behaviors is not significant. |
| [(Sirois et al., 2015)](#Sirois2015) | CS | CA | 339 | College Students | GPS | PSS | S：r=0.32 | Mediation analysis supports that stress plays a role in explaining the association between procrastination and sleep quality, with stress fully mediating the relationship between procrastination and sleep. |
| [(Sirois & Biskas, 2024)](#Sirois2024) | CS | CA | 597 | Adult | AIP-R | PSS | S：r=0.39 | Chronic procrastination indirectly affects self-rated health by increasing stress and reducing healthy behaviors. |
| [(Sirois, 2014)](#Sirois2014) | CS | CA | 145 | College Students | GPS | PSS | S：r=0.23 | Interventions that enhance self-compassion may help reduce stress related to procrastination. |
| [(Stead et al., 2010)](#Stead2010) | CS | CA | 200 | College Students | GPS  PASS | DHS-R | S:r=0.22 | Procrastination and stress work together to have negative effects on mental health and help-seeking behaviors. |
| [(Sun et al., 2024)](#Sun2024) | CS | CN | 1137 | College Students | IPS | Time anxiety scale | A：r=0.49 | Time anxiety in college students not only directly affects their sleep quality but also indirectly influences it through irrational procrastination. Physical activity plays a moderating role. |
| [(Tarman & Sari, 2023)](#Tarman2023) | CS | TR | 242 | College Students | GPS | LSAS | A：r=0.31 | There is a positive association between social anxiety and procrastination. Additionally, mindfulness partially mediates this relationship. |
| [(To et al., 2021)](#To2021) | CS | CN | 154 | College Students | GPS | DASS-21 | S：r=0.06 | Self-control mediates the relationship between stress and procrastination. |
| [(Wu et al., 2025)](#Wu2025) | CS | CN | 451 | College Students | IPS | SAS-SMU | A：r=0.53 | Fear of missing out (FoMO) not only directly predicts online social anxiety but also indirectly affects it through irrational procrastination and media multitasking. |
| [(Yang et al., 2023)](#Yang2023) | CS | CN | 586 | College Students | PASS | PSS-10  PHQ-9 | S:r=0.215  D：r=0.325 | Among college students, physical activity affects depressive symptoms directly and indirectly through the independent and chain mediating effects of perceived stress and academic procrastination. |
| [(Yang et al., 2020)](#Yang2020) | CS | CN | 1004 | College Students | GPS | DASS-21 | S：r=0.29 | Trait procrastination may be a risk factor for smartphone addiction among college students. Additionally, this relationship can be mediated by stress. |
| [(Yang et al., 2023)](#Yang2023Z) | CS | CN | 1217 | College Students | BPC | SRQ-20 | S：r=0.31 | Psychological stress responses are associated with increased bedtime procrastination due to increased smartphone addiction, particularly among young people with higher family cohesion. |
| [(Yang, 2018)](#Yang2018Z) | CS | CN | 475 | College Students | IPS | AEQ | A：r=0.39 | Smartphone use mediates the relationship between self-regulation, academic anxiety, and academic procrastination. |
| [(Yildirim & Demir, 2020)](#Yildirim2020) | CS | TR | 801 | College Students | TPS | AEQ  Test anxiety | A：r=0.22 | Procrastination is positively correlated with test anxiety. |
| [(Zhang et al., 2023)](#Zhang2023) | CS | CN | 698 | College Students | BPS | TAI | A：r=0.345 | Trait anxiety is an important independent predictor of bedtime procrastination and can induce bedtime procrastination directly or indirectly through the influence of self-control. |
| [(Zhang & Zhang, 2022)](#Zhang2022) | CS | CN | 55 | College Students | APS | SLWAI | A：R=0.382 | Academic procrastination can indirectly affect the Flesch-Kincaid grade level of L2 writing through L2 writing anxiety. |
| [(Zhang et al., 2020)](#Zhang2020) | CS | CN | 168 | College Students | GPS | STAI | A：r=0.46 | From a neuroscientific perspective, a positive correlation between trait anxiety and procrastination is observed. |
| [(Zhang et al., 2022)](#Zhangx2022) | CS | CN | 306 | College Students | GPS | DASS-21 | D:r=0.35  A:r=0.31  S:r=0.24 | Academic procrastination has a significant impact on internet addiction, which is formed through the multiple mediating effects of intrusive thoughts and negative emotions. |
| [(Zhu et al., 2023)](#Zhu2023) | CS | CN | 668 | College Students | BPC | DASS-21 | D:r=0.52  A:r=0.41  S:r=0.47 | Smartphone addiction and negative emotions mediate the relationship between the tendency for boredom and bedtime procrastination. |
| [(Zhu et al., 2024)](#Zhu2024) | CS | CN | 665 | Middle School Students | BPC | DASS-21 | D:r=0.41  A:r=0.42 | Bedtime procrastination partially mediates the relationships between parental psychological control and depression, as well as parental psychological control and anxiety. |
| [(Ziaaddini & Alinezhad, 2019)](#Ziaaddini2019) | CS | IR | 242 | Adult | Not | Jop stress | S：r=0.538 | Job stress and job satisfaction significantly impact work-related procrastination among employees in the research organization. |
| [(Maria-Ioanna & Patra, 2022)](#Marialoanna2022) | CS | GR | 681 | College Students | PASS | SCL-R-90 | D:r=0.25  A:r=0.23 | Correlational analyses between academic procrastination and psychopathology indicate that the total score of the Procrastination Assessment Scale for Students (PASS) is positively correlated with psychopathology. |
| [(Maria-Ioanna & Patra, 2022)](#Marialoanna2022) | CS | GR | 182 | College Students | PASS | SCL90 | D:r=0.30  A:r=0.28 | Students who procrastinate, especially those failing to complete tasks, have higher anxiety, more distress, and lower life satisfaction. |
| [(Cui et al., 2021)](#Gui2021) | LS | CN | 1235 | College Students | BPS | PHQ-9 | D:r=0.361 | Bedtime procrastination predicts subsequent depressive symptoms. |
| [(Faure-Carvallo et al., 2025)](#Faure2025) | CS | CN | 910 | College Students | PASS | BSI-18 | D:r=0.282  A:r=0.147 | Neuroticism can lead to procrastination. |
| [(Johansson et al., 2023)](#Johansson2023) | LS | SE | 3525 | College Students | PPS | DDAS21 | D：r=0.13  A：r=0.08 | The severity of procrastination is associated with poorer subsequent mental health outcomes, including depressive, anxiety, and stress symptoms. |
| [(Beutel et al., 2016)](#Beutel2016) | CS | DE | 2527 | All age groups | GPS | PSS  PHQ-4  GAD-7 | D:r=0.36  A：r=0.32  S：r=0.39 | Procrastination is significantly associated with perceived stress, depression, anxiety, fatigue, and decreased life satisfaction in various domains such as work and leisure. |
| [(Sirois, 2007)](#Sirois2007) | CS | Many | 254 | Adult | GPS  AIP  DPS | stress over the past 6 months | S：r=0.13  r=0.18  r=0.17 | Stress fully mediates the relationship between procrastination and health. However, when considering the combined effects of stress and health behaviors, health behaviors do not mediate this relationship. |
| [(Markiewicz & Kaczmarek, 2024)](#Markiewicz2024) | CS | PL | 344 | Middle School Students | IDS | DASS 42 | D：β=0.429  A：β=0.363  S：β=0.392 | The mediating effect of delay in the relationship between depression, stress, and school burnout. |
| [(Wang, 2021)](#Wang2021) | LS | CN | 264 | Middle School Students | API | TAS | A：r=0.314 | Academic procrastination (T1) → Test anxiety (T2) Test anxiety (T1) → N.S. → Academic procrastination (T2) |
| [(Reinecke et al., 2018)](#Reinecke2018) | CS | DE | 1577 | All age groups | GPS | PSS  PHQ4 | D:r=0.34  A：r=0.30  S：r=0.42 | Individuals with high trait procrastination are more susceptible to the negative impacts of internet use in other areas of their lives. |
| [(Jochmann et al., 2024)](#Jochmann2024) | LS | DE | 392 | College Students | PFS-4 | HEI-STRESS  Pss | S=0.20 | Over time, procrastination can lead to symptoms of depression and anxiety, and perceived stress is not a mediator of this effect. |
| [(Sirois & Tosti, 2012)](#Sirois2012) | CS | IT | 339 | College Students | GPS | PSS | S：r=0.32 | The impact of procrastination on stress and health is mediated by mindfulness. |
| [(Monaghan et al., 2024)](#Monaghan2024) | CS | US | 1309 | Adult | PPS | CESD-8 | D:β=0.31 | While age itself is not directly related to procrastination, the likelihood of experiencing depression decreases with age, and this decrease in depression is associated with a lower likelihood of procrastination. |

CS:Cross sectional study; LS:Longitudinal study; SEM:Structural Equation Model Testing; PPS: Pure Procrastination Scale; Bedtime Procrastination Scale; APS:**Academic Procrastination Scale; PASS:**Procrastination Assessment Scale for Students; GPS:General Procrastination Scale; TPS:Tuckman Procrastination Scale; API:Aitken Procrastination Inventory; AIP: Adult Inventory of Procrastination; IPS:Irrational Procrastination Scle; PFS:Procrastination For Student; IDS:Implemental delay scale; DASS:Depression Anxiety Stress Scales; TAS:Test Anxiety Scale; SCL-90:Symptom Checklist-90; AEQ:Academic Emotion Questionnaire; CES-D:Center for Epidemiologic Studies Depression Scale; CTAS:Cognitive Test Anxiety Scale; SGSS:Self-generated stress scale; STAI:State-Trait Anxiety Inventory; PHQ:Patient Health Questionnaire; CES-D:Center for Epidemiologic Studies Depression Scale; PSS:Perceived Stress Scale; BSI-18:Brief Symptom Inventory 18; CESD-8:Center for Epidemiologic Studies Depression Scale-8; SRQ-20:Self-Reporting Questionnaire-20; SLWAL:Second Language Writing Anxiety Inventory; SLAS:Liebowitz Social Anxiety Scale; GHQ-20:General Health Questionnaire; DHS-R:Daily Hassles Scale-Revised; GADQ-IV:Generalized Anxiety Disorder Questionnaire-IV.

Table2 Results of Literature Quality Assessment.

| Studuy | sample representativeness | measurement instruments | Completeness of Results | confounding factors considered | exposure status | Sum |
| --- | --- | --- | --- | --- | --- | --- |
| [(Jamil et al., 2021)](#Jamil2021) | 1 | 1 | 1 | 1 | 1 | 5 |
| [(Deng et al., 2022)](#Deng2022) | 1 | 1 | 1 | 1 | 1 | 5 |
| [(Aftab et al., 2017)](#Aftab2017) | 1 | 1 | 1 | 1 | 1 | 5 |
| [(Ion Albulescu et al., 2024)](#Ion2024) | 0 | 1 | 1 | 1 | 1 | 4 |
| [(Ariani & Susilo, 2018)](#Ariani) | 0 | 1 | 1 | 0 | 1 | 3 |
| [(Babayiğit et al., 2024)](#Babayigit) | 0 | 1 | 1 | 0 | 1 | 3 |
| [(Barel et al., 2023)](#Barel2023) | 0 | 1 | 1 | 1 | 1 | 4 |
| [(Cao et al., 2025)](#Cao2025) | 1 | 1 | 1 | 1 | 1 | 5 |
| [(Cong et al., 2024)](#Cong2023) | 0 | 1 | 1 | 1 | 1 | 4 |
| [(Constantin et al., 2018)](#Constant2018) | 0 | 1 | 1 | 1 | 1 | 4 |
| [(Eisenbeck et al., 2019)](#Eisenbeck2019) | 0 | 1 | 1 | 1 | 1 | 4 |
| [(Elhai et al., 2021)](#Elhai2021) | 0 | 1 | 1 | 1 | 1 | 4 |
| [(Fan et al., 2024)](#Fan2024) | 1 | 1 | 1 | 1 | 1 | 5 |
| [(Fernie et al., 2016)](#Fernie2016) | 0 | 1 | 1 | 1 | 1 | 4 |
| [(Maji et al., 2024)](#Maji2024) | 0 | 1 | 1 | 0 | 1 | 3 |
| [(Flett et al., 2016)](#Flett2016) | 0 | 1 | 1 | 0 | 1 | 3 |
| [(Folgado-Alufre et al., 2022)](#Folgado2022) | 0 | 1 | 1 | 1 | 1 | 4 |
| [(Gadosey et al., 2024)](#Gadosey2024) | 1 | 1 | 1 | 1 | 1 | 5 |
| [(Gadosey et al., 2024)](#Gadosey2024（2）) | 1 | 1 | 1 | 0 | 1 | 4 |
| [(Geng et al., 2021)](#Geng2021) | 0 | 1 | 1 | 0 | 1 | 3 |
| [(Ghattas & El-Ashry, 2024)](#Ghattas2024) | 1 | 1 | 1 | 1 | 1 | 5 |
| [(Glick et al., 2014)](#Glick2014) | 0 | 1 | 1 | 0 | 1 | 3 |
| [(Gong et al., 2021)](#Gong2021) | 0 | 1 | 1 | 1 | 1 | 4 |
| [(Guo et al., 2023)](#Guo2023) | 1 | 1 | 1 | 1 | 1 | 5 |
| [(Hayat et al., 2024)](#Hayat2024) | 0 | 1 | 1 | 1 | 1 | 4 |
| [(Haycock et al., 1998)](#Haycock1998) | 0 | 1 | 0 | 0 | 1 | 2 |
| [(He et al., 2025)](#He2025) | 1 | 1 | 1 | 1 | 1 | 5 |
| [(Hernandez et al., 2019)](#Hernandez2019) | 1 | 1 | 1 | 1 | 1 | 5 |
| [(Hou & Hu, 2023)](#Hou2023) | 1 | 1 | 1 | 1 | 1 | 5 |
| [(Hutchison et al., 2018)](#Hutchison2018) | 0 | 1 | 0 | 1 | 1 | 3 |
| [(Jia et al., 2021)](#Jia2021) | 0 | 1 | 1 | 1 | 1 | 4 |
| [(Jin et al., 2024)](#Jin2024) | 0 | 1 | 1 | 1 | 1 | 4 |
| [(Khalid et al., 2019)](#Khaid2019) | 0 | 1 | 1 | 0 | 1 | 3 |
| [(Kinik & Odaci, 2020)](#Kinik2020) | 1 | 1 | 1 | 1 | 1 | 5 |
| [(Ko & Chang, 2019)](#Ko2019) | 0 | 1 | 1 | 1 | 1 | 4 |
| [(Krispenz et al., 2019)](#Krispenz2019) | 0 | 1 | 1 | 1 | 1 | 4 |
| [(Kühnel et al., 2023)](#Kühnel2023) | 0 | 1 | 1 | 0 | 1 | 3 |
| [(Lee et al., 2025)](#Lee2025) | 0 | 1 | 1 | 1 | 1 | 4 |
| [(Li et al., 2024)](#Li2024) | 1 | 1 | 1 | 1 | 1 | 5 |
| [(Li et al., 2024)](#Lix2024) | 0 | 1 | 1 | 0 | 1 | 3 |
| [(Liu et al., 2024)](#Liu2024) | 1 | 1 | 1 | 0 | 1 | 4 |
| [(Liu & Li, 2024)](#Liu20242) | 1 | 1 | 1 | 1 | 1 | 5 |
| [(Ma et al., 2022)](#Ma2022) | 0 | 1 | 1 | 1 | 1 | 4 |
| [(Malik & Ashraf, 2019)](#Mailk2019) | 0 | 1 | 1 | 1 | 1 | 4 |
| [(Meng et al., 2024)](#Meng2024) | 1 | 1 | 1 | 1 | 1 | 5 |
| [(Petwal et al., 2021)](#Petwal2021) | 0 | 1 | 1 | 0 | 1 | 3 |
| [(Przepiorka et al., 2023)](#Przepiorka2023) | 0 | 1 | 1 | 1 | 1 | 4 |
| [(Qiao et al., 2023)](#Qiao2023) | 1 | 1 | 1 | 1 | 1 | 5 |
| [(Ragusa et al., 2023)](#Ragusa2023) | 1 | 1 | 1 | 1 | 1 | 5 |
| [(Reinecke et al., 2018)](#Peinecke2018) | 1 | 1 | 1 | 1 | 1 | 5 |
| [(Rezaei-Gazki et al., 2024)](#Rezaei2024) | 0 | 1 | 1 | 1 | 1 | 4 |
| [(Rogowska & Cincio, 2024)](#Rogowska2024) | 0 | 1 | 1 | 1 | 1 | 4 |
| [(Roshanisefat et al., 2021)](#Roshanisefat2021) | 0 | 1 | 1 | 0 | 1 | 3 |
| [(Saddler & Sacks, 1993)](#Saddler1993) | 0 | 1 | 0 | 0 | 1 | 2 |
| [(Shang et al., 2023)](#Shang2023) | 0 | 1 | 1 | 1 | 1 | 4 |
| [(Sirois et al., 2023)](#Sirois2023) | 1 | 1 | 1 | 1 | 1 | 5 |
| [(Sirois et al., 2015)](#Sirois2015) | 0 | 1 | 1 | 0 | 1 | 3 |
| [(Sirois & Biskas, 2024)](#Sirois2024) | 1 | 1 | 1 | 1 | 1 | 5 |
| [(Sirois, 2014)](#Sirois2014) | 0 | 1 | 1 | 0 | 1 | 3 |
| [(Stead et al., 2010)](#Stead2010) | 0 | 1 | 1 | 0 | 1 | 3 |
| [(Sun et al., 2024)](#Sun2024) | 1 | 1 | 1 | 1 | 1 | 5 |
| [(Tarman & Sari, 2023)](#Tarman2023) | 0 | 1 | 1 | 1 | 1 | 4 |
| [(To et al., 2021)](#To2021) | 0 | 1 | 1 | 0 | 1 | 3 |
| [(Wu et al., 2025)](#Wu2025) | 0 | 1 | 1 | 1 | 1 | 4 |
| [(Yang et al., 2023)](#Yang2023) | 0 | 1 | 1 | 0 | 1 | 3 |
| [(Yang et al., 2020)](#Yang2020) | 1 | 1 | 1 | 1 | 1 | 5 |
| [(Yang et al., 2023)](#Yang2023Z) | 1 | 1 | 1 | 1 | 1 | 5 |
| [(Yang, 2018)](#Yang2018Z) | 0 | 1 | 1 | 1 | 1 | 4 |
| [(Yildirim & Demir, 2020)](#Yildirim2020) | 0 | 1 | 1 | 1 | 1 | 4 |
| [(Zhang et al., 2023)](#Zhang2023) | 0 | 1 | 1 | 1 | 1 | 4 |
| [(Zhang & Zhang, 2022)](#Zhang2022) | 0 | 1 | 1 | 1 | 1 | 4 |
| [(Zhang et al., 2020)](#Zhang2020) | 0 | 1 | 1 | 0 | 1 | 3 |
| [(Zhang et al., 2022)](#Zhangx2022) | 0 | 1 | 1 | 1 | 1 | 4 |
| [(Zhu et al., 2023)](#Zhu2023) | 1 | 1 | 1 | 1 | 1 | 5 |
| [(Zhu et al., 2024)](#Zhu2024) | 1 | 1 | 1 | 1 | 1 | 5 |
| [(Ziaaddini & Alinezhad, 2019)](#Ziaaddini2019) | 0 | 1 | 1 | 0 | 1 | 3 |
| [(Maria-Ioanna & Patra, 2022)](#Marialoanna2022) | 0 | 1 | 1 | 0 | 1 | 3 |
| [(Cui et al., 2021)](#Gui2021) | 1 | 1 | 1 | 1 | 1 | 5 |
| [(Faure-Carvallo et al., 2025)](#Faure2025) | 1 | 1 | 1 | 1 | 1 | 5 |
| [(Johansson et al., 2023)](#Johansson2023) | 1 | 1 | 1 | 1 | 1 | 5 |
| [(Beutel et al., 2016)](#Beutel2016) | 1 | 1 | 1 | 1 | 1 | 5 |
| [(Sirois, 2007)](#Sirois2007) | 0 | 1 | 1 | 1 | 1 | 4 |
| [(Markiewicz & Kaczmarek, 2024)](#Markiewicz2024) | 0 | 1 | 1 | 1 | 1 | 4 |
| [(Wang, 2021)](#Wang2021) | 0 | 1 | 1 | 1 | 1 | 4 |
| [(Reinecke et al., 2018)](#Reinecke2018) | 1 | 1 | 1 | 1 | 1 | 5 |
| [(Jochmann et al., 2024)](#Jochmann2024) | 1 | 1 | 1 | 1 | 1 | 5 |
| [(Sirois & Tosti, 2012)](#Sirois2012) | 0 | 1 | 1 | 1 | 1 | 4 |
| [(Monaghan et al., 2024)](#Monaghan2024) | 1 | 1 | 1 | 1 | 1 | 5 |

Literature search strategy(2025.4)

WEB OF SCIENSE

("PROCRASTINATION" OR "PROCRASTINATION BEHAVIOUR" OR " PROCRASTINATION TENDENCY" OR "DELAYING BEHAVIOUR") AND ("ANXIETY" OR "ANXIETY DISORDER" OR "GENERALIZED ANXIETY DISORDER" OR "SOCIAL ANXIETY DISORDER" OR "PANIC DISORDER" OR "DEPRESSION" OR "MAJOR DEPRESSIVE DISORDER" OR "CLINICAL DEPRESSION" OR "DYSTHYMIA" OR "STRESS " OR "STRESSOR" OR "STRESS RESPONSE" OR "OCCUPATIONAL STRESS " OR "CHRONIC STRESS")

EBSCO

(DE "PROCRASTINATION" OR DE "PROCRASTINATION BEHAVIOR" OR DE "PROCRASTINATION TENDENCY") AND (DE "ANXIETY" OR DE "ANXIETY DISORDERS" OR DE "GENERALIZED ANXIETY DISORDER" OR DE "SOCIAL ANXIETY DISORDER" OR DE "PANIC DISORDER" OR DE "DEPRESSION" OR DE "MAJOR DEPRESSIVE DISORDER" OR DE "CLINICAL DEPRESSION" OR DE "DYSTHYMIA" OR DE "STRESS" OR DE "STRESSORS" OR DE "STRESS RESPONSE" OR DE "OCCUPATIONAL STRESS" OR DE "CHRONIC STRESS")

PUBMED

(PROCRASTINATION OR "PROCRASTINAN BEHAVIOR" OR "PROCRASTINATION TENDENCY" OR "DELAYING BEHAVIOR") AND (ANXIETY OR "ANXIETY DISORDER" OR "GENERALIZED ANXIETY DISORDER" OR "SOCIAL ANXIETY DISORDER" OR "PANIC DISORDER" OR DEPRESSION OR "MAJOR DEPRESSIVE DISORDER" OR "CLINICAL DEPRESSION" OR "DYSTHYMIA" OR STRESS OR "STRESSOR" OR "STRESS RESPONSETIO" OR "OCCUPATIONAL STRESS" OR "CHRONIC STRESS")

PROQUEST

TITLE(("PROCRASTINATION" OR "PROCRASTINATION BEHAVIOR" OR "PROCRASTINATION TENDENCY" OR "DELAYING BEHAVIOR"))

AND TITLE(("ANXIETY" OR "ANXIETY DISORDER" OR "GENERALIZED ANXIETY DISORDER" OR "SOCIAL ANXIETY DISORDER" OR "PANIC DISORDER" OR DEPRESSION OR "MAJOR DEPRESSIVE DISORDER" OR "CLINICAL DEPRESSION" OR "DYSTHYMIA" OR STRESS OR "STRESSOR" OR "STRESS RESPONSE" OR "OCCUPATIONAL STRESS" OR "CHRONIC STRESS"))

SCOPUS

TITLE-ABS-KEY ( ( PROCRASTINATION OR "PROCRASTINATION BEHAVIOR" OR "PROCRASTINATION TENDENCY" OR "DELAYING BEHAVIOR" ) AND ( ANXIETY OR "ANXIETY DISORDER" OR "GENERALIZED ANXIETY DISORDER" OR "SOCIAL ANXIETY DISORDER" OR "PANIC DISORDER" OR DEPRESSION OR "MAJOR DEPRESSIVE DISORDER" OR "CLINICAL DEPRESSION" OR "DYSTHYMIA" OR STRESS OR "STRESSOR" OR "STRESS RESPONSE" OR "OCCUPATIONAL STRESS" OR "CHRONIC STRESS" ) ) AND ( LIMIT-TO ( DOCTYPE , "AR" ) )

One low-quality study was in the anxiety model, and one was in the depression model. After excluding low-quality studies, the comparison of effect sizes τ² and I² showed…

Table 3 Comparative Analysis of Anxiety Model Sensitivity

| Anxiety | k | Fisher’s Z (95 % CI) | τ² | I² |
| --- | --- | --- | --- | --- |
| ALL | 50 | 0.352 (0.317–0.386) | 0.0124 | 90.60% |
| After exclusion | 49 | 0.352 (0.317–0.387) | 0.0126 | 90.86% |

Table 4 Comparative Analysis of Depression Model Sensitivity

| Depression | k | Fisher’s Z (95 % CI) | τ² | I² |
| --- | --- | --- | --- | --- |
| ALL | 32 | 0.369 (0.333-0.405) | 0.0081 | 85.82% |
| After exclusion | 31 | 0.370 (0.334–0.407) | 0.0083 | 86.44% |

Sensitivity analysis excluding extreme sample sizes

Table 5 Sensitivity analysis after excluding sample size outliers.

| Depression | k | Fisher’s Z (95 % CI) | τ² | I² |
| --- | --- | --- | --- | --- |
| ALL | 32 | 0.369 (0.333-0.405) | 0.0081 | 85.82% |
| After excluding extreme values | 26 | 0.381 (0.344-0.417) | 0.0066 | 79.53% |

Depression, Anxiety, Stress One-by-One Exclusion Method, Testing the Impact of Individual Studies on Heterogeneity. Numeric coding, consistent with the order of study names in the forest plot in the main text.

Figure 1

Depression

Figure 2

Anxiety

Figure 3

Stress

**Reference**

Jamil, L., Ashouri, A., Zamirinejad, S., & Mahaki, B. (2021). Investigating the structural model of procrastination based on transdiagnostic factors. *Medical Journal of the Islamic Republic of Iran*. <https://doi.org/10.47176/mjiri.35.120>

Deng, Y., Ye, B., & Yang, Q. (2022). COVID-19 Related Emotional Stress and Bedtime Procrastination Among College Students in China: A Moderated Mediation Model. Nature and Science of Sleep, Volume 14, 1437–1447. <https://doi.org/10.2147/nss.s371292>

Aftab, S., Klibert, J., Holtzman, N., Qadeer, K., & Aftab, S. (2017). Schemas mediate the link between procrastination and depression: Results from the United States and Pakistan. Journal of Rational - Emotive and Cognitive - Behavior Therapy, 35(4), 329–345. Scopus. <https://doi.org/10.1007/s10942-017-0263-5>

Ion Albulescu, Labar, A.-V., Manea, A.-D., & Stan, C. (2024). The mediating role of cognitive test anxiety on the relationship between academic procrastination and subjective wellbeing and academic performance. Frontiers in Public Health, 12. <https://doi.org/10.3389/fpubh.2024.1336002>

Ariani, D., & Susilo, Y. (2018). Why Do It Later? Goal Orientation, Self-efficacy, Test Anxiety, on Procrastination. ECPS - Educational Cultural and Psychological Studies, 17. <https://doi.org/10.7358/ecps-2018-017-wahy>

Babayiğit, O., Büyükkalaycı, F. N., & Altun, S. (2024). The interplay of academic procrastination, self-generated stress, and self-reported bruxism among medical and dental students: a cross-sectional study. BMC Psychology, 12(1). <https://doi.org/10.1186/s40359-024-02105-w>

Barel, E., Shahrabani, S., Mahagna, L., Massalha, R., Colodner, R., & Tzischinsky, O. (2023). State Anxiety and Procrastination: The Moderating Role of Neuroendocrine Factors. Behavioral Sciences, 13(3), 204. <https://doi.org/10.3390/bs13030204>

Cao, C., Chen, D., & Zhou, Y. (2025). Perceived stress and academic procrastination among higher vocational nursing students: the mediating roles of positive and negative emotions. BMC Nursing, 24(1). <https://doi.org/10.1186/s12912-024-02672-8>

Cong, T., Kuang, Y., Bao, Y., & Yu, S. (2024). Effects of perceived academic stress on sleep quality among chinese college students: Mediating effects of social comparison, bedtime procrastination, and the protective role of emotion regulation. Current Psychology, 43(40), 31327–31342. Scopus. <https://doi.org/10.1007/s12144-024-06707-w>

Constantin, K., English, M. M., & Mazmanian, D. (2018). Anxiety, depression, and procrastination among students: Rumination plays a larger mediating role than worry. Journal of Rational - Emotive and Cognitive - Behavior Therapy, 36(1), 15–27. Scopus. <https://doi.org/10.1007/s10942-017-0271-5>

Eisenbeck, N., Carreno, D. F., & Ucles-Juarez, R. (2019). From psychological distress to academic procrastination: Exploring the role of psychological inflexibility. Journal of Contextual Behavioral Science, 13, 103–108. <https://doi.org/10.1016/j.jcbs.2019.07.007>

Elhai, J. D., Sapci, O., Yang, H., Amialchuk, A., Rozgonjuk, D., & Montag, C. (2021). Objectively-measured and self-reported smartphone use in relation to surface learning, procrastination, academic productivity, and psychopathology symptoms in college students. Human Behavior and Emerging Technologies, 3(5), 912–921. Scopus. <https://doi.org/10.1002/hbe2.254>

Fan, J., Cheng, Y., Tang, M., Huang, Y., & Yu, J. (2024). The mediating role of ego depletion in the relationship between state anxiety and academic procrastination among university students. Scientific Reports, 14(1), 15568. MEDLINE. <https://doi.org/10.1038/s41598-024-66293-6>

Fernie, B. A., McKenzie, A.-M., Nikcevic, A. V., Caselli, G., & Spada, M. M. (2016). The contribution of metacognitions and attentional control to decisional procrastination. Journal of Rational-Emotive and Cognitive-Behavior Therapy, 34(1), 1–13. Scopus. <https://doi.org/10.1007/s10942-015-0222-y>

Maji, S., Sinha, S., Chaturmohta, A., & Sharma, S. N. (2024). Punishment sensitivity and depression: The serially mediating role of procrastination and academic satisfaction. Current Psychology, 43(20), 18552–18560. Scopus. <https://doi.org/10.1007/s12144-024-05660-y>

Flett, A. L., Haghbin, M., & Pychyl, T. A. (2016). Procrastination and Depression from a Cognitive Perspective: An Exploration of the Associations Among Procrastinatory Automatic Thoughts, Rumination, and Mindfulness. JOURNAL OF RATIONAL-EMOTIVE AND COGNITIVE-BEHAVIOR THERAPY, 34(3), 169–186. Psychology Collection. <https://doi.org/10.1007/s10942-016-0235-1>

Folgado-Alufre, M., Serrano-Mendizábal, M., Miragall, M., Desdentado, L., García-Ubiedo, L., & Baños, R. M. (2022). The protective role of intrinsic motivation, self-efficacy, and self-regulation strategies against procrastination and statistics anxiety. Statistica Applicata, 34(2). Scopus. <https://doi.org/10.26398/IJAS.0034-009>

Gadosey, C. K., Schnettler, T., Scheunemann, A., Baeulke, L., Thies, D. O., Dresel, M., Fries, S., Leutner, D., Wirth, J., & Grunschel, C. (2024). Vicious and virtuous relationships between procrastination and emotions: An investigation of the reciprocal relationship between academic procrastination and learning-related anxiety and hope. European Journal of Psychology of Education, 39(3), 2005–2031. APA PsycInfo®. <https://doi.org/10.1007/s10212-023-00756-8>

Gadosey, C. K., Turhan, D., Wenker, T., Kegel, L. S., Bobe, J., Thomas, L., Buhlmann, U., Fries, S., & Grunschel, C. (2024). Relationship between the intraindividual interplay of negative and positive exam-related emotions and the behavioral-emotional dimensions of academic procrastination. Current Psychology, 43(40), 31476–31494. Scopus. <https://doi.org/10.1007/s12144-024-06719-6>

Geng, Y., Gu, J., Wang, J., & Zhang, R. (2021). Smartphone addiction and depression, anxiety: The role of bedtime procrastination and self-control. Journal of Affective Disorders, 293(null), 415–421. APA PsycInfo®. <https://doi.org/10.1016/j.jad.2021.06.062>

Ghattas, A. H. S., & El-Ashry, A. M. (2024). Perceived academic anxiety and procrastination among emergency nursing students: The mediating role of cognitive emotion regulation. BMC Nursing, 23(1), 1–11. Scopus. <https://doi.org/10.1186/s12912-024-02302-3>

Glick, D. M., Millstein, D. J., & Orsillo, S. M. (2014). A preliminary investigation of the role of psychological inflexibility in academic procrastination. Journal of Contextual Behavioral Science, 3(2), 81–88. Scopus. <https://doi.org/10.1016/j.jcbs.2014.04.002>

Gong, Z., Wang, L., & Wang, H. (2021). Perceived stress and internet addiction among chinese college students: Mediating effect of procrastination and moderating effect of flow. Frontiers in Psychology, 12, 7. APA PsycInfo®. <https://doi.org/10.3389/fpsyg.2021.632461>

Guo, M., Cao, Y., & Hu, X. (2023). Chinese students’ filial piety beliefs and procrastination in mathematics learning: The mediating role of academic emotions. Frontiers in Psychology, 14. Scopus. <https://doi.org/10.3389/fpsyg.2023.1050259>

Hayat, A. A., Kiani, H., Ramezani, G., & Keshavarzi, M. H. (2024). Examining the mediating role of test anxiety in the relationship between procrastination and self-handicapping of students. Strides in Development of Medical Education Journal, 21(1), 129–137. Scopus. <https://doi.org/10.22062/sdme.2024.199184.1302>

Haycock, L. A., McCarthy, P., & Skay, C. L. (1998). Procrastination in college students: The role of self-efficacy and anxiety. Journal of Counseling and Development : JCD, 76(3), 317–324. Education Database; Psychology Collection; Research Library. <https://doi.org/10.1002/j.1556-6676.1998.tb02548.x>

He, Q., Wu, H., Meng, X., & Li, C. (2025). The relationship between perceived stress and bedtime procrastination among Chinese college students: A moderated mediation model. Frontiers in Psychiatry, 16, 1547389. <https://doi.org/10.3389/fpsyt.2025.1547389>

Hernandez, C., Rivera Ottenberger, D., Moessner, M., Crosby, R. D., & Ditzen, B. (2019). Depressed and swiping my problems for later: The moderation effect between procrastination and depressive symptomatology on internet addiction. Computers in Human Behavior, 97, 1–9. <https://doi.org/10.1016/j.chb.2019.02.027>

Hou, X., & Hu, J. (2023). Depression and bedtime procrastination: Chain mediation of brooding and perceived stress. Heliyon, 9(12), e22672. Scopus. <https://doi.org/10.1016/j.heliyon.2023.e22672>

Hutchison, T. E., Penney, A. M., & Crompton, J. E. (2018). Procrastination and anxiety: Exploring the contributions of multiple anxiety-related disorders. Current Issues in Personality Psychology, 6(2), 122–129. APA PsycInfo®. <https://doi.org/10.5114/cipp.2018.73054>

Jia, J., Wang, L.-L., Xu, J.-B., Lin, X.-H., Zhang, B., & Jiang, Q. (2021). Self-handicapping in chinese medical students during the COVID-19 pandemic: The role of academic anxiety, procrastination and hardiness. Frontiers in Psychology, 12. Scopus. <https://doi.org/10.3389/fpsyg.2021.741821>

Jin, Y., Zhou, W., Zhang, Y., Yang, Z., & Hussain, Z. (2024). Smartphone distraction and academic anxiety: The mediating role of academic procrastination and the moderating role of time management disposition. Behavioral Sciences, 14(9), 820. Publicly Available Content Database. <https://doi.org/10.3390/bs14090820>

Khalid, A., Zhang, Q., Wang, W., Ghaffari, A. S., & Pan, F. (2019). The relationship between procrastination, perceived stress, saliva alpha-amylase level and parenting styles in chinese first year medical students. Psychology Research and Behavior Management, 12(null), 489–498. Scopus. <https://doi.org/10.2147/PRBM.S207430>

Kinik, O., & Odaci, H. (2020). Effects of dysfunctional attitudes and depression on academic procrastination: Does self-esteem have a mediating role? British Journal of Guidance & Counselling, 48(5), 638–649. APA PsycInfo®. <https://doi.org/10.1080/03069885.2020.1780564>

Ko, C.-Y. A., & Chang, Y. (2019). Investigating the relationships among resilience, social anxiety, and procrastination in a sample of college students. Psychological Reports, 122(1), 231–245. APA PsycInfo®. <https://doi.org/10.1177/0033294118755111>

Krispenz, A., Gort, C., Schueltke, L., & Dickhaeuser, O. (2019). How to reduce test anxiety and academic procrastination through inquiry of cognitive appraisals: A pilot study investigating the role of academic self-efficacy. Frontiers in Psychology, 10(AUG), 1917. APA PsycInfo®. <https://doi.org/10.3389/fpsyg.2019.01917>

Kühnel, J., Bledow, R., & Kuonath, A. (2023). Overcoming procrastination: Time pressure and positive affect as compensatory routes to action. Journal of Business and Psychology, 38(4), 803–819. Scopus. <https://doi.org/10.1007/s10869-022-09817-z>

Lee, T., Cho, E., Ahmed, O., Ahn, J., Bang, Y. R., Chung, S., & Park, J. (2025). The impact of depression on bedtime procrastination in high school students in pandemic era: The mediating roles of conscientiousness and emotional stability. International Journal of Behavioral Medicine. APA PsycInfo®. <https://doi.org/10.1007/s12529-025-10351-4>

Li, C., Qu, S., & Ren, K. (2024). The role of self-esteem and depression in the relationship between physical activity and academic procrastination among Chinese undergraduate students: A serial mediation model. Psychology Research And Behavior Management, 17(null), 3721–3729. Scopus. <https://doi.org/10.2147/PRBM.S477659>

Li, G., Xiong, Z., & Lin, P. (2024). Anxiety and academic procrastination in deaf and hard of hearing college students: A moderated mediation model. Behavioral Sciences, 14(12), 1219. Publicly Available Content Database. <https://doi.org/10.3390/bs14121219>

Li, X., Xu, Y., Li, G., Ning, L., Xie, X., Shao, C., Liu, C., & Yang, X. (2024). The relationship between academic procrastination and anxiety symptoms among medical students during the COVID-19 pandemic: Exploring the moderated mediation effects of resilience and social support. BMC Psychiatry, 24(1), 1–11. Coronavirus Research Database; Psychology Collection; Publicly Available Content Database. <https://doi.org/10.1186/s12888-024-06202-3>

Liu, N., Wang, J., & Zang, W. (2024). The impact of sleep determination on procrastination before bedtime: The role of anxiety. International Journal Of Mental HEALTH Promotion, 26(5), 377–387. Scopus. <https://doi.org/10.32604/ijmhp.2024.047808>

Liu, Q., & Li, J. (2024). A one-year longitudinal study on the mediating role of problematic TikTok use and gender differences in the association between academic stress and academic procrastination. Humanities & Social Sciences Communications, 11(1), 1132. Coronavirus Research Database; Publicly Available Content Database; Research Library. <https://doi.org/10.1057/s41599-024-03654-6>

Ma, Y., Yang, X. M., Hong, L., & Tang, R. J. (2022). The influence of stress perception on academic procrastination in postgraduate students: The role of self-efficacy for self-regulated learning and self-control. International Journal of Digital Multimedia Broadcasting, 2022. Scopus. <https://doi.org/10.1155/2022/6722805>

Malik, J. A., & Ashraf, M. (2019). Academic stress predicted by academic procrastination among young adults: Moderating role of peer influence resistance. Journal of the Liaquat University of Medical and Health Sciences, 18(1), 65–70. Scopus. <https://doi.org/10.22442/jlumhs.191810603>

Meng, S., Zhang, Y., Tang, L., Zhang, M., Tang, W., Onyebuchi, N., Han, Y., Han, S., Li, B., Tong, W., & Ge, X. (2024). The effects of mobile phone addiction on bedtime procrastination in university students: The masking effect of physical activity and anxiety. BMC Psychology, 12(1), 1–11. Scopus. <https://doi.org/10.1186/s40359-024-01899-z>

Petwal, P., Sudhir, P. M., & Mehrotra, S. (2021). Procrastination and self-compassion in individuals with anxiety disorders. Telangana Journal of Psychiatry, 7(1), 22–28. <https://doi.org/10.4103/tjp.tjp_20_21>

Przepiorka, A., Blachnio, A., & Cudo, A. (2023). Procrastination and problematic new media use: The mediating role of future anxiety. Current Psychology, 42(7), 5169–5177. Scopus. <https://doi.org/10.1007/s12144-021-01773-w>

Qiao, Z., Wu, Y., Xie, Y., Qiu, X., Chen, L., Yang, J., Pan, H., Gu, S., Yang, X., Hu, X., Wei, P., Zhao, J., Qu, Y., Zhou, J., Bu, T., & Yang, Y. (2023). The chain mediating roles of anxiety and depression in the relationship between the effects of the COVID-19 pandemic and procrastination in adolescents: A longitudinal study. BMC Public Health, 23(1), 1–8. Coronavirus Research Database; Publicly Available Content Database. <https://doi.org/10.1186/s12889-023-16605-8>

Ragusa, A., Gonzalez-Bernal, J., Trigueros, R., Caggiano, V., Navarro, N., Minguez-Minguez, L. A., Obregon, A., I., & Fernandez-Ortega, C. (2023). Effects of academic self-regulation on procrastination, academic stress and anxiety, resilience and academic performance in a sample of spanish secondary school students. Frontiers in Psychology, 14, 1073529. Scopus. <https://doi.org/10.3389/fpsyg.2023.1073529>

Reinecke, L., Meier, A., Beutel, M. E., Schemer, C., Stark, B., Woelfling, K., & Mueller, K. W. (2018). The relationship between trait procrastination, internet use, and psychological functioning: Results from a community sample of german adolescents. Frontiers in Psychology, 9. <https://doi.org/10.3389/fpsyg.2018.00913>

Rezaei-Gazki, P., Ilaghi, M., & Masoumian, N. (2024). The triangle of anxiety, perfectionism, and academic procrastination: Exploring the correlates in medical and dental students. BMC MEDICAL EDUCATION, 24(1), 1–7. Education Database; Publicly Available Content Database. <https://doi.org/10.1186/s12909-024-05145-3>

Rogowska, A. M., & Cincio, A. (2024). Procrastination mediates the relationship between problematic TikTok use and depression among young adults. Journal of Clinical Medicine, 13(5), 1247. Publicly Available Content Database. <https://doi.org/10.3390/jcm13051247>

Roshanisefat, S., Azizi, S. M., & Khatony, A. (2021). Investigating the relationship of test anxiety and time management with academic procrastination in students of health professions. Education Research International, 2021. Education Database; Publicly Available Content Database; Research Library. <https://doi.org/10.1155/2021/1378774>

Saddler, C. D., & Sacks, L. A. (1993). Multidimensional perfectionism and academic procrastination: Relationships with depression in university students. Psychological Reports, 73(3, Pt 1), 863–871. APA PsycInfo®. <https://doi.org/10.2466/pr0.1993.73.3.863>

Shang, Z., Cao, Y., Cui, Z., & Zuo, C. (2023). Positive delay? The influence of perceived stress on active procrastination. South African Journal of Business Management, 54(1). Scopus. <https://doi.org/10.4102/sajbm.v54i1.3988>

Sirois, F. M., Stride, C. B., & Pychyl, T. A. (2023). Procrastination and health: A longitudinal test of the roles of stress and health behaviours. British Journal of Health Psychology, 28(3), 860–875. Scopus. <https://doi.org/10.1111/bjhp.12658>

Sirois, F. M., van Eerde, W., & Argiropoulou, M. I. (2015). Is procrastination related to sleep quality? Testing an application of the procrastination–health model. Cogent Psychology, 2(1), 1074776. Scopus. <https://doi.org/10.1080/23311908.2015.1074776>

Sirois, F. M., & Biskas, M. (2024). Procrastination and health in nurses: Investigating the roles of stress, health behaviours and social support. International Journal of Environmental Research and Public Health, 21(7), 898. Coronavirus Research Database; Publicly Available Content Database. <https://doi.org/10.3390/ijerph21070898>

Sirois, F. M. (2014). Procrastination and stress: Exploring the role of self-compassion. Self and Identity, 13(2), 128–145. APA PsycInfo®. <https://doi.org/10.1080/15298868.2013.763404>

Stead, R., Shanahan, M. J., & Neufeld, R. W. J. (2010). “I’ll go to therapy, eventually”: Procrastination, stress and mental health. Personality and Individual Differences, 49(3), 175–180. APA PsycInfo®. <https://doi.org/10.1016/j.paid.2010.03.028>

Sun, Z., Gao, X., & Ren, P. (2024). The relationship between time anxiety and college students’ sleep quality: The mediating role of irrational procrastination and the moderating effect of physical activity. Frontiers in Psychology, 15, 1410746. Scopus. <https://doi.org/10.3389/fpsyg.2024.1410746>

Tarman, G. Z., & Sari, B. A. (2023). The mediating role of mindfulness on social anxiety and procrastination. International Journal of Mental Health and Addiction, 21(2), 1013–1024. Scopus. <https://doi.org/10.1007/s11469-021-00637-5>

To, P.-Y.-L., Lo, B.-C.-Y., Ng, T.-K., Wong, B.-P.-H., & Choi, A.-W.-M. (2021). Striving to avoid inferiority and procrastination among university students: The mediating roles of stress and self-control. International Journal of Environmental Research and Public Health, 18(11), 5570. Publicly Available Content Database. <https://doi.org/10.3390/ijerph18115570>

Wang, Q., Kou, Z., Du, Y., Wang, K., & Xu, Y. (2022). Academic procrastination and negative emotions among adolescents during the COVID-19 pandemic: The mediating and buffering effects of online-shopping addiction. Frontiers in Psychology, 12, 789505. <https://doi.org/10.3389/fpsyg.2021.789505>

Wu, W., Zhang, J., & Jo, N. (2025). Fear of missing out and online social anxiety in university students: Mediation by irrational procrastination and media multitasking. Behavioral Sciences, 15(1), 84. Publicly Available Content Database. <https://doi.org/10.3390/bs15010084>

Xiong, Z., Li, G., Chen, J., & Peng, L. (2024). The impact of executive dysfunction on anxiety in hearing-impaired college students: Smartphone addiction as a mediator and academic procrastination as a moderator. Psychology Research and Behavior Management, 17(null), 2853–2863. Scopus. <https://doi.org/10.2147/PRBM.S470293>

Yang, L., Liu, Z., Shi, S., Dong, Y., Cheng, H., & Li, T. (2023). The mediating role of perceived stress and academic procrastination between physical activity and depressive symptoms among Chinese college students during the COVID-19 pandemic. International Journal of Environmental Research and Public Health, 20(1), 773. Scopus. <https://doi.org/10.3390/ijerph20010773>

Yang, X., Wang, P., & Hu, P. (2020). Trait procrastination and mobile phone addiction among chinese college students: A moderated mediation model of stress and gender. Frontiers in Psychology, 11, 9. APA PsycInfo®. <https://doi.org/10.3389/fpsyg.2020.614660>

Yang, X., Zhu, J., & Hu, P. (2023). Perceived social support and procrastination in college students: A sequential mediation model of self-compassion and negative emotions. Current Psychology, 42(7), 5521–5529. <https://doi.org/10.1007/s12144-021-01920-3>

Yang, Z., Huang, J., Li, Z., Xu, H., & Guo, C. (2023). The effect of smartphone addiction on the relationship between psychological stress reaction and bedtime procrastination in young adults during the COVID-19 pandemic. <https://research.ebsco.com/linkprocessor/plink?id=83b8a70b-dbd0-3c77-8e32-9a2825ba4422>

Yang, Z. (2018). An exploration of problematic smartphone use among chinese university students: Associations with academic anxiety, academic procrastination, self-regulation and subjective well-being. Journal of Behavioral Addictions, 7, 166–166.

Yildirim, F. B., & Demir, A. (2020). Self-handicapping among university students: The role of procrastination, test anxiety, self-esteem, and self-compassion. Psychological Reports, 123(3), 825–843. APA PsycInfo®. <https://doi.org/10.1177/0033294118825099>

Zhang, C., Meng, D., Zhu, L., Ma, X., Guo, J., Fu, Y., Zhao, Y., Xu, H., & Mu, L. (2023). The effect of trait anxiety on bedtime procrastination: The mediating role of self-control. International Journal of Behavioral Medicine, 30(2), 260–267. Scopus. <https://doi.org/10.1007/s12529-022-10089-3>

Zhang, C., & Zhang, W. (2022). The impact of academic procrastination on second language writing: The mediating role of L2 writing anxiety. Frontiers in Psychology, 13, 851120. APA PsycInfo®. <https://doi.org/10.3389/fpsyg.2022.851120>

Zhang, R., Chen, Z., Xu, T., Zhang, L., & Feng, T. (2020). The overlapping region in right hippocampus accounting for the link between trait anxiety and procrastination. Neuropsychologia, 146, 8. APA PsycInfo®. <https://doi.org/10.1016/j.neuropsychologia.2020.107571>

Zhang, X., Chen, K., Wang, M., & Chen, C. (2022). The relationship between academic procrastination and internet addiction in college students: The multiple mediating effects of intrusive thinking and depression-anxiety-stress. Psychology, 13(4), 591–606. <https://doi.org/10.4236/psych.2022.134040>

Zhu, Y., Liu, J., Wang, Q., Huang, J., Li, X., & Liu, J. (2023). Examining the association between boredom proneness and bedtime procrastination among chinese college students: A sequential mediation model with mobile phone addiction and negative emotions. Psychology Research & Behavior Management, 16, 4329–4340. Academic Search Ultimate. <https://doi.org/10.2147/PRBM.S431615>

Zhu, Y., Wang, Q., Liu, J., & Huang, J. (2024). Parental psychological control and depression, anxiety among adolescents: The mediating role of bedtime procrastination and moderating role of neuroticism. <https://research.ebsco.com/linkprocessor/plink?id=f520ac6e-da24-3599-8c6c-0a2f8a86271e>

Ziaaddini, M., & Alinezhad, H. (2019). The mediator role of procrastination in the association between job characteristics and job stress among employees of shahid rajaee customs office, bandar abbas, iran (2017). Journal of Occupational Health and Epidemiology, 8(4), 221–225. Scopus. <https://doi.org/10.29252/johe.8.4.221>

Maria-Ioanna, A., & Patra, V. (2022). The role of psychological distress as a potential route through which procrastination may confer risk for reduced life satisfaction. Current Psychology, 41(5), 2860–2867. Scopus. <https://doi.org/10.1007/s12144-020-00739-8>

Cui, G., Yin, Y., Li, S., Chen, L., Liu, X., Tang, K., & Li, Y. (2021). Longitudinal relationships among problematic mobile phone use, bedtime procrastination, sleep quality and depressive symptoms in chinese college students: A cross-lagged panel analysis. BMC Psychiatry, 21(1). Scopus. <https://doi.org/10.1186/s12888-021-03451-4>

Faure-Carvallo, A., Nieto-Fernandez, S., Calderon, C., & Gustems, J. (2025). Relationship between procrastination, time management, personality, and psychological distress in higher education. Journal of Further and Higher Education. <https://doi.org/10.1080/0309877X.2025.2459852>

Johansson, F., Rozental, A., Edlund, K., Cote, P., Sundberg, T., Onell, C., Rudman, A., & Skillgate, E. (2023). Associations between procrastination and subsequent health outcomes among university students in Sweden. JAMA Network Open, 6(1), E2249346. Scopus. <https://doi.org/10.1001/jamanetworkopen.2022.49346>

Beutel, M. E., Klein, E. M., Aufenanger, S., Braehler, E., Dreier, M., Mueller, K. W., Quiring, O., Reinecke, L., Schmutzer, G., Stark, B., & Woelfling, K. (2016). Procrastination, distress and life satisfaction across the age range—A german representative community study. PLOS One, 11(2), e0148054. Scopus. <https://doi.org/10.1371/journal.pone.0148054>

Sirois, F. M. (2007). “I’ll look after my health, later”: A replication and extension of the procrastination-health model with community-dwelling adults. Personality and Individual Differences, 43(1), 15–26. Scopus. <https://doi.org/10.1016/j.paid.2006.11.003>

Markiewicz, K., & Kaczmarek, B. L. J. (2024). Implemental delay as a mediator of the relationship between depression, anxiety, stress and school burnout. PLOS One, 19(12). Scopus. <https://doi.org/10.1371/journal.pone.0316082>

Wang, Y. (2021). Academic procrastination and test anxiety: A cross-lagged panel analysis. Journal of Psychologists and Counsellors in Schools, 31(1), 122–129. Education Database; Psychology Collection. <https://doi.org/10.1017/jgc.2020.29>

Reinecke, L., Meier, A., Aufenanger, S., Beutel, M. E., Dreier, M., Quiring, O., Stark, B., Wölfling, K., & Müller, K. W. (2018). Permanently online and permanently procrastinating? The mediating role of internet use for the effects of trait procrastination on psychological health and well-being. New Media and Society, 20(3), 862–880. Scopus. <https://doi.org/10.1177/1461444816675437>

Jochmann, A., Gusy, B., Lesener, T., & Wolter, C. (2024). Procrastination, depression and anxiety symptoms in university students: A three-wave longitudinal study on the mediating role of perceived stress. <https://research.ebsco.com/linkprocessor/plink?id=9a6445ec-aca6-3a72-b193-b9c85f264490>

Sirois, F. M., & Tosti, N. (2012). Lost in the moment? An investigation of procrastination, mindfulness, and well-being. Journal of Rational - Emotive and Cognitive - Behavior Therapy, 30(4), 237–248. Scopus. <https://doi.org/10.1007/s10942-012-0151-y>

Monaghan, C., Avila-Palencia, I., Han, S. D., & Power, J. M. (2024). Procrastination, depressive symptomatology, and loneliness in later life. Aging and Mental Health, 28(9), 1270–1277. Scopus. <https://doi.org/10.1080/13607863.2024.2345781>
